# Charge Regulation during Amyloid Formation of α-Synuclein

**DOI:** 10.1021/jacs.1c01925

**Published:** 2021-05-17

**Authors:** Tinna Pálmadóttir, Anders Malmendal, Thom Leiding, Mikael Lund, Sara Linse

**Affiliations:** † Department of Biochemistry and Structural Biology, Lund University, P.O. Box 124, 221 00, Lund, Sweden; ‡ Department of Science and Environment, Chemistry, Roskilde University, Roskilde, Denmark; § Department of Theoretical Chemistry, Lund University, Lund, Sweden; ∥ LINXS - Lund Institute of Advanced Neutron and X-ray Science, Lund University, Lund, Sweden

## Abstract

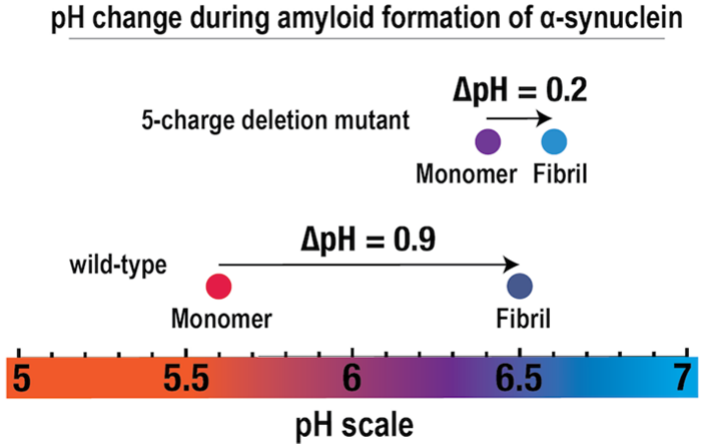

Electrostatic interactions play crucial roles in protein function. Measuring p*K*_a_ value perturbations upon complex formation or self-assembly of e.g. amyloid fibrils gives valuable information about the effect of electrostatic interactions in those processes. Site-specific p*K*_a_ value determination by solution NMR spectroscopy is challenged by the high molecular weight of amyloid fibrils. Here we report a pH increase during fibril formation of α-synuclein, observed using three complementary experimental methods: pH electrode measurements in water; colorimetric changes of a fluorescent indicator; and chemical shift changes for histidine residues using solution state NMR spectroscopy. A significant pH increase was detected during fibril formation in water, on average by 0.9 pH units from 5.6 to 6.5, showing that protons are taken up during fibril formation. The pH upshift was used to calculate the average change in the apparent p*K*_a_^ave^ value of the acidic residues, which was found to increase by at least 1.1 unit due to fibril formation. Metropolis Monte Carlo simulations were performed on a comparable system that also showed a proton uptake due to fibril formation. Fibril formation moreover leads to a significant change in proton binding capacitance. Parallel studies of a mutant with five charge deletions in the C-terminal tail revealed a smaller pH increase due to fibril formation, and a smaller change (0.5 units on average) in the apparent p*K*_a_^ave^ values of the acidic residues. We conclude that the proton uptake during the fibril formation is connected to the high density of acidic residues in the C-terminal tail of α-synuclein.

## 1 Introduction

Seven out of the 20 amino acids, Asp, Glu, Lys, Arg, His, Tyr, Cys, as well as the N and C termini, contain ionizable groups, giving rise to the acid/base properties of proteins.^1^ In 1917 Sørensen pioneered the research on protein titrations, demonstrating that egg albumin is an ampholyte with both acid and base properties.^2^ The ionizable side chain groups (as well as the terminal groups) are the major contributors to the electrostatic interactions of proteins, affecting the structure, solubility, stability, and function, such as the kinetic properties.^3−8^

Proteins are large heteropolymeric polyelectrolytes that can carry many charges simultaneously. The sum of the charged state of the ionizable side chain groups, which in turn depends on interactions and the p*K*_a_ values of the individual groups, defines the net charge of the protein. Linderstøm-Lang discovered that the protein’s net charge can affect and perturb the p*K*_a_ values of its titratable groups.^9,10^ Since then, p*K*_a_ perturbations of amino acid residues in proteins have been studied extensively and are frequently found to play important roles in protein function and folding.^11^ The quantification of the perturbations of p*K*_a_ values of individual residues from their intrinsic p*K*_a_ values thus provide valuable information about the their environment and conformational transitions upon folding and binding.^12^

The p*K*_a_ values of individual side chain groups can be down-shifted or up-shifted relative to their intrinsic p*K*_a_ values as measured for model peptides^4,8,13,14^ (Table 1). The three main factors that contribute to p*K*_a_ perturbations are Coulomb interactions (charge–charge interactions), charge–dipole interactions, and desolvation.^8,15^ p*K*_a_ values of ionizable groups free in solutions are generally different from the p*K*_a_ value of the same group buried in a protein interior due to differences in the polarity of the environment (desolvation). The pH dependence of enzyme catalysis can be tailored by modifications of p*K*_a_ values and altered Coulomb interactions, leading to changes in the electrostatic environment within the active site.^16^ The p*K*_a_ values of ionizable groups within an enzyme’s active sites change as a result of altered electrostatic interactions with other adjacent ionizable groups.^15^ Studying p*K*_a_ perturbations within enzymes’ active sites has given an increased understanding of the role of ionizable residues within enzymatic reactions.^15,17−20^

**Table 1:** Comparison of the Intrinsic p*K*_a_ Values of Aspartic Acid and Glutamic Acid to the p*K*_a_ Values Observed in Small Model Peptides, Folded Proteins, and α-Synuclein Monomer

	Asp	Glu
Intrinsic p*K*_a_^a^	4.0	4.4
p*K*_a_ in alanine pentapeptide^b^	3.9	4.3
p*K*_a_ in random coil model peptide^c^	3.9 ± 0.1	4.3 ± 0.1
p*K*_a_ in folded proteins^b^	3.5 ± 1.2	4.2 ± 0.9
p*K*_a_ in full length α-synuclein monomer^d^	4.0 ± 0.4	4.4 ± 0.3
p*K*_a_ in N-terminal and NAC region of α-synuclein^d^	3.6 ± 0.05	4.2 ± 0.1
p*K*_a_ in C-terminal tail of α-synuclein^d^	4.2 ± 0.3	4.6 ± 0.2

a From ref (42).

b From ref (8).

c From ref (43); p*K*_a_ of the ionizable amino acid residue x of the c-H-Gly-Gly-X-l-Ala-OH peptide.

d Average of published values from reference in absence of salt.^30^

Large p*K*_a_ value shifts are often found for ionizable residues with low solvent accessible surface area (SASA). A study by Pahari and Alexov (2019) showed that the p*K*_a_ values of Asp with less than 40% SASA range from 0.5 to 9.9. For Glu residues with 30% or less SASA, they found p*K*_a_ values lower than 2.6 and higher than 6.^15,21^ Charged residues on protein surfaces, with greater SASA, are also commonly found to shift, but the degree of perturbations is generally less than 2 units.^15^ In the same study Pahari and Alexov found that 68% of the Asp residues have more than 40% SASA, and p*K*_a_ values range between approximately 2 and 5.3. Similarly, about 75% of the Glu residues have more than 40% SASA and p*K*_a_ values that range from 2.6 to 6.^21^ High p*K*_a_ values, up to 6.5, are found for Glu residues in a Ca^2+^-binding site of S100G, also known as calbindin D_9k_.^22^ Perturbations of residues on protein surfaces are generally due to Coulomb interactions (charge–charge interactions).^23^ Proteins are evolved for function, which requires a compromise between solubility and stability, which are affected by protein net charge in opposite ways. Electrostatic interaction between groups on protein surfaces^24^ as well as within unstructured proteins contribute to the protein stability, where favorable interactions can increase the stability of the protein state, and very commonly, unfavorable electrostatic repulsion decreases the protein stability.^25,26^

The p*K*_a_ values of individual titratable groups within proteins can be perturbed due to interactions with nearby residues, or due to interactions with neighboring biomolecules or ligands. For example, electrostatic interactions play important roles in the stability and formation of large self-assembling protein structures, such as the tobacco mosaic virus protein and amyloid fibrils.^6,27,13^

**Figure 1:**
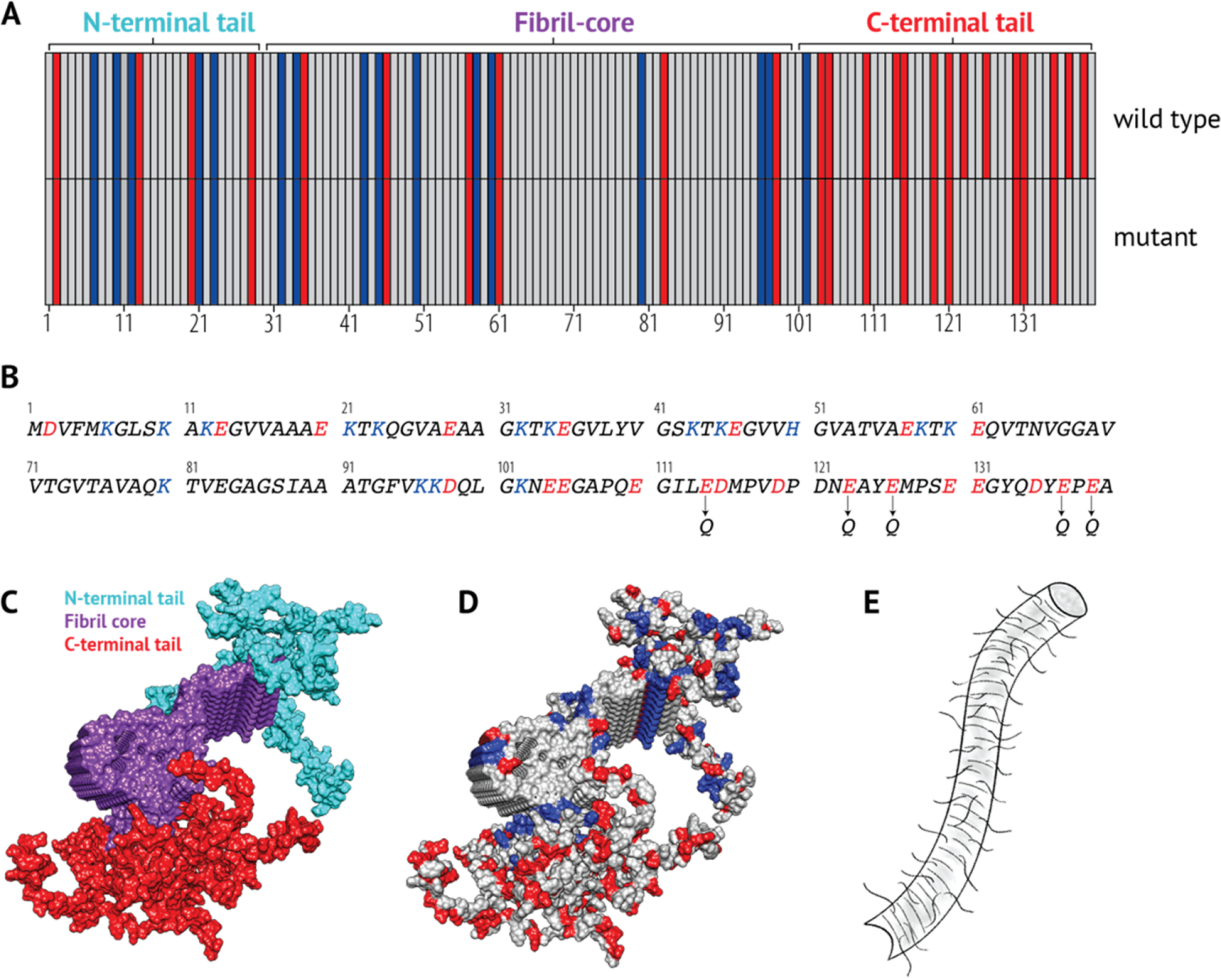
Sequence and structural properties of α-synuclein fibrils. (A) Distribution of acidic (red) and basic (blue) residues within the α-synuclein sequence. The sequence can be divided into three regions according to how it forms amyloid-fibrils: the fibril core (residues 29–100); the N-terminus (residues 1–28); and the C-terminal tail (101–140) (according to 2NOA.pdb). The C-terminal tail of the 5Q mutant is shown in comparison to the wild-type, where five acidic residues have been mutated to noncharged polar residues, making the tail less acidic. (B) Sequence of α-synuclein. Acidic residues are colored red, and basic residues are colored blue. The positions of the mutations (E114Q+E123Q+E126Q+E137Q+E139Q) of the 5Q mutant are shown below the sequence. (C) Proto-filament structure of full-length α-synuclein (pdb: 2N0A(31)) colored according to the 3 regions. The fibril core, purple; the N-terminal tail, cyan; the C-terminal tail, red. (D) Proto-filament structure of α-synuclein colored according to acidic (Red: Glu, Asp) and basic (Blue: Lys, His) residues. (E) A simplified illustration of α-synuclein fibrils, with the extending tails not being a part of the fibril core.

α-Synuclein is a 140 residue long and intrinsically disordered protein, for which the formation of amyloid fibrils and their accumulation into Lewy bodies is associated with Parkinson’s disease.^28^ The protein sequence is commonly divided into three regions: the N-terminal (amphipathic) region, the central hydrophobic region, also called the NAC region, and the C-terminal (acidic) tail.^29,30^ However, the boundaries of the regions may vary depending on the context, as suggested from solved structures.^31^ Here, the protein will be divided into the N-terminal tail, the hydrophobic fibril core, and the C-terminal tail (Figure 1) based on how the monomeric protein arranges into a fibrillar structure as well as the distribution of charge residues within the sequence.^31,30,29^ The last 40 residues of α-synuclein—referred to as the C-terminal tail—are the focus of this project. The tail is highly acidic, consisting of 15 acidic groups (including the C-terminus), and is highly charged, with a net charge of −12 at physiological pH.^32^ Previous studies have indicated that the C-terminal tail is not a part of the fibril core and remains unstructured in both the monomeric and fibrillar form.^31,30,33−36^ The C-terminal tail is believed to extend from the fibril core like a polymer brush at conditions where it is highly negatively charged.^33^

The mechanism of aggregation of α-synuclein into amyloid fibrils is highly dependent on pH, the process is dominated by surface catalyzed secondary nucleation below pH 6.0 and is much slower at higher pH. The pH dependence is believed to be linked to the high acidity of the C-terminal tail.^37,38^ Truncation of the C-terminal tail, as well as charge-screening, has been found to affect the aggregation kinetics of α-synuclein.^37,39−41^ Further studies on the effect of the acidic residues in the tail of α-synuclein on the amyloid formation are important in order to gain a deeper understanding of the pH dependence of α-synuclein aggregation and the role of electrostatic interactions in α-synuclein amyloid formation.

The p*K*_a_ values of the acidic residues of the monomeric α-synuclein have previously been measured using NMR spectroscopy and were found to be higher in the C-terminal tail compared to the first 100 residues (Table 1).^30^ These p*K*_a_ value perturbations of the acidic residues in the C-terminal tail could be explained by the high charge density and the close proximity between negative charges within the tail.^37,30^Table 1 gives a comparison of intrinsic p*K*_a_ values of the acidic residues, Asp and Glu, to their p*K*_a_ values measured within small model peptides, folded proteins, and α-synuclein monomer.

In this study, the effect of amyloid formation on the p*K*_a_ values of the acidic residues in the C-terminal tail of α-synuclein was examined. The goal was to compare the p*K*_a_ values of α-synuclein monomers and fibrils, which could provide a better understanding of the role of electrostatic interactions and the effect of the C-terminal tail on the fibril formation process. The main questions were as follows: Do the p*K*_a_ values of the acidic residues become upshifted during amyloid formation? If so, is that a result of the high density of acidic residues in the unstructured C-terminal tail? These questions were addressed using three complementary experimental methods to monitor the change in pH upon fibril formation in water as well as through Metropolis Monte Carlo computer simulations. The data for wild-type α-synuclein were compared to a mutant lacking five acidic residues in the C-terminal tail (Figure 1a,b), to provide a relative measure of the effect of the negative charges in the tail.

## 2 Experimental Section

### 2.1 Protein Expression

*Escherichia coli* (*E*. *coli*) BL21* pLysS Ca^2+^ competent cells were transformed with pET-3a-plasmid containing the gene for wild-type human α-synuclein (not N-acetylated) with *E. coli*-optimized codons and an ATG start codon (purchased from GenScript, Piscataway, New Jersey; see Supporting Information). The same was done for the mutated α-synuclein used in this study. 0.7 μL of plasmid (100 ng/μL) was carefully mixed with 30 to 40 μL of Ca^2+^-competent cells and kept on ice for 30 to 60 min, and the sample was heat-pulsed for 45 s at 42 °C and placed on ice for 10 min. The transformed cells were spread on sterile LB agar plates supplemented with chloramphenicol (30 μg/mL) and ampicillin (50 μg/mL) and incubated at 37 °C overnight (ON). The plate was stored at 5 °C for 8 h before a small single colony was selected for inoculation of 50 mL culture (LB medium with chloramphenicol (30 μg/mL) and ampicillin (50 μg/mL)) which was incubated ON at 37 °C with shaking. Next morning, 5 mL overnight culture was transferred to each 500 mL day culture (LB medium with chloramphenicol (30 μg/mL) and ampicillin (50 μg/mL)) in 2.5 L baffled flasks. The day culture was incubated at 37 °C with continuous shaking at 125 rpm. The optical density at 600 nm (OD_600 nm_) was followed, and when it had reached approximately 0.9–1.0, protein expression was induced with 100 μg/mL isopropyl thio-β-d-galactoside (IPTG). The cells were harvested 4 h after induction, by centrifugation at 6000*g* for 12 min at 4 °C (JA 8.100 rotor). The supernatant was disposed, and the cells obtained from total of 4 L culture were combined, mixed with 25 mL of water, and frozen. Before harvesting, 1 mL samples were taken from the cultures for testing the expression by SDS-PAGE.

Expression of ^15^N labeled wild-type α-synuclein was done in M9 minimal medium. In this case, 3 mL of overnight culture in LB medium with ^15^NH_4_Cl as the sole nigrogen source, (supplemented with chloramphenicol (30 μL/mL) and ampicillin (50 μL/mL)) were added to 50 mL of middle-day culture in M9 minimal medium supplemented with chloramphenicol (30 μL/mL) and ampicillin (50 μL/mL). When OD_600 nm_ had reached 1.0, then 50 mL of middle-day-culture were transferred to 450 mL of M9 minimal medium (supplemented with chloramphenicol (30 μL/mL) and ampicillin (50 μL/mL)). When *D*_600 nm_ of the 500 mL culture had reached 0.7–0.9, the expression was induced with 100 μg/mL IPTG. The cells were harvested 4 h after induction as above.

### 2.2 Purification

Cell pellet from 8 L culture was thawed in 100–120 mL of cold buffer A (10 mM Tris/HCl, 1 mM EDTA, pH 7.5) and placed on ice. The volume of the buffer was approximately 5 times the volume of the cell pellet. The pellet was sonicated on ice, using pulse sonication (1 s on, 1 s off) until the mixture remained homogeneous. After sonication the sample was centrifuged for 10 min at 15000*g* at 4 °C (JA 25.50 rotor). The supernatant was collected (approximately 130 mL). The supernatant was poured into an equal volume of boiling buffer A. In order to precipitate *E. coli* proteins, the sample was heated to 85 °C. The sample was placed on ice and continuously stirred while cooling down. The sample was centrifuged at 15 000*g* for 10 min at 4 °C. The supernatant was collected and further purified by ion-exchange chromatography.

The first ion-exchange chromatography step was performed using 100 g of wet diethylaminoethyl (DEAE) cellulose in a column with a diameter of 3.5 cm. Before packing of the column, the resin was washed at least two times with Milli-Q water and two times with buffer A, or until the pH was measured to be around 7.5. All buffers were kept cold, and the purification was performed in a cold room. The column was equilibrated with 100 mL of buffer A. The sample (about 260 mL) was loaded onto the column which was then washed with 100 mL of buffer A. The sample was eluted at a flow rate of 1 mL/min using linear 0–0.5 M NaCl gradient in buffer A (total gradient volume of 1.4 L). SDS-PAGE or agarose gel electrophoresis was used to determine which fractions contained α-synuclein. The fractions containing α-synuclein were pooled, diluted 50:50 with buffer A, and further purified on a DEAE sephacel column (60 g of wet resin in a column with diameter of 2.3 cm). The second ion-exchange chromatography step was performed in the same way as above for the DEAE cellulose column. The absorbance at 280 nm was measured and further analyzed with SDS-PAGE to determine which fraction contained α-synuclein (see appendix). The fractions containing α-synuclein and no detectable impurities were pooled and stored as 1 mL aliquots at −20 °C. The concentration (in the range of 1–3 mg/mL) was determined by absorbance at 280 nm, using an extinction coefficient of ε = 5800 M^–1^cm^–1^. All buffers and milli-Q water were filtrated and degassed before use. The corrected mass was confirmed, and the purity was tested using MALDI-TOF and MALDI-TOF-TOF mass spectrometry.

The expression and purification of the α-synuclein 5Q mutant was performed in the same way as for the wild-type, except for the sample being eluted using a linear 0–0.35 M NaCl gradient in buffer instead of 0–0.5 M, in both the first and second ion-exchange chromatography step.

### 2.3 Preparation of Samples

Prior to each experiment, monomeric α-synuclein was isolated by size exclusion chromatography (SEC) in the desired buffer. When buffer free protein was needed, the sample was subsequently loaded onto a desalting column in order to exchange the buffer for milli-Q water in order to get α-synuclein in water.

#### 2.3.1 Size-Exclusion Chromatography

To further concentrate the sample, the fractions containing α-synuclein were lyophilized and dissolved in a smaller volume of 6 M guanidinium hydrochloride. Prior to loading the sample onto the SEC column, the sample was incubated at room temperature for at least 1 h to ensure that the protein was properly dissolved. The sample was injected onto a Superdex 75 Increase 10/300 GL (GE Healthcare) column using a fast protein liquid chromatography (FPLC) system (Bio-RAD, BIOLOgic Duo Flow, USA). The sample was eluted at 0.7 mL/min in the desired buffer. The center of the monomeric peak (1–1.5 mL) was collected into low-binding tubes (Genuine Axygen Quality). The absorbance at 280 nm was used to follow the elution of the monomeric α-synuclein. The concentration was determined from the absorbance at 280 nm using an extinction coefficient of ε = 5800 M^–1^ cm^–1^. All buffer solutions were prepared fresh, degassed, and filtered before usage.

#### 2.3.2 Desalting Column

To obtain α-synuclein in pure water, without any buffer, a HiTrap Desalting column (GE Healtcare) was used to separate α-synuclein from buffer. The sample (1 mL sample collected from precious SEC chromatography step) was injected onto the desalting column and eluted at 1 mL/min in degassed and filtrated Milli-Q water using a FPLC system. The center of the protein peak was collected into low-binding tubes and used for further experiments. The concentration was determined from the absorbance at 280 nm using an extinction coefficient of ε = 5800 M^–1^ cm^–1^.

### 2.4 Fibril Formation Kinetics

Monomeric α-synuclein was prepared the same day as the experiment started and kept on ice until the beginning of the experiment. The α-synuclein fibrils were formed by incubating 100 μL of monomeric α-synuclein in a nontreated 96 well polystyrene black plate (3631 Costar) under quiescent conditions. The plate had a transparent bottom and was sealed with a transparent SealPlate film in order to avoid evaporation. The fibril formation was indirectly measured by supplementing the samples with 20 μM Thioflavin T (ThT), and the fluorescence was monitored over time using a Fluostar plate reader (BMG Labtech, Offenburg, Germany) at 37 °C without shaking using excitation and emission wavelengths of 440 and 480 nm, respectively.

### 2.5 Confirming the Presence of Amyloid Fibrils

#### 2.5.1 Far-UV Circular Dichroism (CD) Spectroscopy

The Far-UV CD spectra were recorded between 260 and 190 nm at 20 °C in a 0.1 mm quartz cuvette using a Jasco J-815 CD spectrometer. The spectra were acquired with a scanning speed of 50 nm/min, continuous scanning mode, digital integration time per data point (D.I.T) of 8 s, and sensitivity set to standard. The data were averaged over three accumulations.

#### 2.5.2 ANS Fluorescence

The fluorescence of the optical probe, 8-Anilino-1-napthalenesulfonate (ANS), is highly sensitive to the environment and can easily increase about 20 times in a nonpolar or rigid environment.^44^ Binding of ANS to hydrophobic patches of amyloid fibrils results in a significant increase in fluorescence intensity.^45^ For validation of the presence of fibrils, ANS (10 μM) was supplemented to aliquots of the samples of interest, before and after fibril formation. The samples were excited at 395 nm, and the emission spectra were recorded from 410 nm to 600 (PerkinElmer luminescence Spectrometer LS-50B, UK).

### 2.6 Detection of pH Changes

#### 2.6.1 Detection of pH Change Using pH Meter

After isolation of monomeric samples in pure water using a desalting column, the samples were diluted to 20 or 70 μM α-synuclein in filtrated Milli-Q water. The pH of an aliquot from the sample was measured with an Orion star A211 pH meter and an Orion ROSS Combination pH Micro Electrode. The rest of the sample was incubated at 37 °C in a 2 mL low binding-tube (Genuine Axygen Quality), with continuous stirring at 700 rpm using a micro stirring bar (8 mm × 1.5 mm, Polytetrafluoroethylene (PTFE) coated, strong Alnico V magnetic core, round smooth surface). The presence of fibrils was confirmed using CD spectroscopy and/or ANS fluorescence using a withdrawn aliquot. The pH of the sample containing amyloid fibrils was measured using the same pH electrode.

#### 2.6.2 Continuous pH Measurement during Fibril Formation Using Probe Drum

The pH was measured continuously using an Orion star A211 pH meter connected to an Orion ROSS Combination pH Micro Electrode and a Probe Drum instrument (Probation Laboratories Sweden AB). A 1 mL aliquot of 70 μM α-synuclein prepared in water was placed in a Quartz SUPRASIL (HellmaAnalytics) cuvette with a path length of 10 mm. A micro stirring bar (8 mm × 1.5 mm, PTFE coated) was added to the cuvette, and the sample was stirred at 237 rpm (setting nr. 4) at 37 °C and pH was measured continuously.

In some of these experiments, the pH, static light scattering, and ThT fluorescence were measured simultaneously using Probe Drum. The aggregation and pH measurements were performed the same way as explained above, except for including ThT in the sample (20 μM). Spectra were recorded from 300 to 730 nm at each time point. The ThT fluorescence was measured using a UV-light source of 392 nm, and the average fluorescence signal between 475 and 485 nm was plotted against time. The static light scattering was measured at 90° angle using a laser of 635 nm, and the average signal between 633 and 638 nm was plotted against time.

#### 2.6.3 pH Detected Using pH Indicator

Monomeric α-synuclein was isolated in a weak buffer system consisting of 0.5 mM 2-(*N*-morpholino)ethanesulfonic acid (MES), 12.5 μM resazurin, and 0.02% NaN_3_ at pH 5.5. Part of the monomeric sample was stored at 5 °C to prevent fibril formation; this was done for later color comparison between monomeric and fibrillar sample. The other part of the sample was incubated in 2 mL low-binding tubes at 37 °C with stirring at 900 rpm (IKA IKAMAG REO Magnetic Stirrer) with a micro stirring bar (8 mm × 1.5 mm, PTFE coated). After 1 day of incubation, the color of the incubated sample was compared, at room temperature, to the color of the monomeric sample. In addition, an aliquot of the samples was taken before and after the incubation at 37 °C, and the pH was measured using a pH electrode. Also far-UV CD (260–190 nm) spectroscopy was used to verify whether the samples were monomeric or fibrillar by checking what secondary structural component was dominant in the sample.

#### 2.6.4 pH Change Detected Using NMR Spectroscopy by Using Histidine as a pH Sensor

The pH change during fibril formation was detected by comparing the chemical shifts of the δ2 proton in the imidazole ring of histidine before and after the fibrillation of α-synuclein. 70 μM α-synuclein was isolated in 99.9% D_2_O, without any buffer by using a two-step preparation, including SEC on a Superdex 75 column followed by SEC on a G25 desalting column. The sample was split into two parts, one to be kept monomeric and one to become fibrillar. The monomeric sample was stored on ice until the measurements started. The fibrillar sample was prepared by incubating the sample at 37 °C, with stirring at 700 rpm (IKA IKAMAG REO Magnetic Stirrer) with a micro stirring bar (0.8 cm × 0.3 cm, PTFE coated) for 2 days. The presence of fibrils was confirmed using far-UV CD spectroscopy. Solution state 1D ^1^H (proton) NMR spectra were acquired for the monomeric and fibrillar samples in 100% D_2_O at 300 K using an Agilent VNMRS DirectDrive spectrometer, operating at a ^1^H frequency of 499.9 MHz, equipped with a triple resonance probe. The ^1^H NMR spectra were acquired using a single-90°-pulse experiment. The water signal was suppressed by presaturation. A total of 256 transients of 16 k data points spanning a spectral width of 16 ppm were collected.

### 2.7 Measurement of Monomer Concentration after Fibril Formation

Wild-type and 5Q mutant were prepared in water as explained in sections 2.3.1 and 2.3.2. The samples were diluted to 20 μM. An aliquot of the sample was stored on ice until further analysis with SDS-PAGE (10–20% Tris-Tricine gel). The samples of both variants were split into three tubes each, containing a 500 μL sample. The samples were incubated at 37 °C in 2 mL low-binding tubes (Genuine Axygen Quality), with continuous stirring at 700 rpm using a micro stirring bar (8 mm × 1.5 mm) for 2 days. The presence of fibrils was confirmed using CD spectroscopy, of a withdrawn aliquot. Two-step separation of fibrils and soluble monomers was performed as follows: 300 μL of each sample were centrifuged for 10 min at 14600*g* and room temperature in a 1.5 mL low-binding tube. Next, 150 μL of the supernatant were removed from the top of the sample and transferred to another 1.5 mL low-binding tube. The supernatant was centrifuged again for 25 min at 14 600*g* at room temperature. Thereafter, 50 μL were taken of the supernatant for further analysis with SDS-PAGE. CD spectroscopy was used to verify the separation of monomers and fibrils, by taking spectra of both supernatant and resuspended pellet.

Monomer (that had been stored on ice) and the supernatants of wild-type and 5Q mutant were loaded onto a polyacrylamide gel. The concentration of the monomer in equilibrium with fibrils was calculated by analyzing the intensity of the gel bands, using the program ImageJ. The intensity of each band was compared to that of a 6 μM monomeric sample.

### 2.8 Cryogenic Transmission Electron Microscopy (Cryo-TEM)

The fibrils were formed in a 96-well plate as previously explained. The protein concentration was 35 μM. The samples were supplemented with 20 μM ThT, and its fluorescence was recorded to ensure that the sample contained fibrils and had reached the final plateau before collection at 55 h. The fibrils tend to stick to the bottom of the well, and therefore the sample was collected by carefully scraping the bottom of the well, using an Eppendorf tip. Before the sample was frozen, it was carefully pipetted up and down. All samples were treated in the same way. To ensure a stable temperature and avoiding loss of solution during sample preparation, a controlled environment vitrification system was used. Samples were prepared as thin liquid films (<300 nm thick) on glow-discharged treated lacey carbon film coated copper grids and plunged into liquid ethane at −180 °C. In this way the original microstructures are preserved as we can avoid component segmentation and rearrangement in addition to water crystallization as the samples are vitrified. Samples were stored under liquid N_2_ until measured and then transferred using an Oxford CT3500 cryoholder and its workstation into the electron microscope (Philips CM120 BioTWIN Cryo) equipped with a postcolumn energy filter (Gatan GIF100). An acceleration voltage of 120 kV was used, and images were recorded digitally with a CCD camera under low electron dose conditions.

### 2.9 Diffusion Ordered NMR Spectroscopy

Diffusion ordered NMR spectroscopy (DOSY) was used to characterize the size of the NMR detected molecular species. Here the signal intensity of the NAC region of the protein monomer was followed as a function of gradient strength in both the absence and presence of fibrils. Measurements were done in 100% D_2_O at 300 K on a Bruker Avance III HD 800 spectrometer (Bruker Biospin, Rheinstetten, Germany), operating at a ^1^H frequency of 799.9 MHz, equipped with a 5 mm cold probe using the ledbpgp2s1d sequence with stimulated echo and bipolar gradient pulses.^46^ Spectra were recorded at four gradient strengths with a diffusion delay of 100 ms and a gradient pulse duration of 4 ms. A total of 32 transients of 32 k data points spanning a spectral width of 16 ppm were collected. The intensities were integrated over the regions 7.29–6.68, 4.59–3.70, 3.10–2.11, 2.00–1.28, and 0.90–0.72 ppm and normalized to the intensity at the lowest gradient strength.

### 2.10 Two-Dimensional Heteronuclear NMR Spectroscopy

2D ^15^N–^1^H heteronuclear single quantum coherence (HSQC) spectra were recorded in 5% D_2_O/95% H_2_O at 300 K on the same Bruker Avance III HD 800 spectrometer, using the hsqcetgpsi2 sequence. A total of 2048 × 128 data points spanning 14 × 30 ppm in the ^1^H and ^15^N dimensions were collected.

### 2.11 Molecular Modeling: Metropolis Monte Carlo Simulations

To study the ionization states of α-synuclein monomers and fibrils, constant pH Metropolis Monte Carlo simulations were performed using a coarse-grained protein model where protonation states are allowed to fluctuate according to their chemical environment. The model has previously been used to study α-synuclein^41^ and presented in detail elsewhere (https://dx.doi.org/ 10/mrv). The use of constant pH in the simulations explicitly accounts for any coupling between titrating groups. All residues of the protein are included in the simulations. Amino acid residues are treated as single beads either connected with harmonic bonds (N-term 1–28, C-term 101–140) or kept fixed (residues 29–100) according to their mass center positions from the fibril structure found in PDB 2N0A. Amino acid beads interact through a combined Lennard–Jones and screened Coulomb potential where the latter uses a Bjerrum length of 7 Å and a screening length corresponding to 0.5 mM 1:1 salt. Residues participating in acid/base equilibria are subject to Monte Carlo charge swap moves mimicking (de)protonation and associated with a trial energy contribution of Δ*u* = *k*_B_*T*(pH – p*K*_a_^0^) + Δ*u*_elec_, where *k*_B_ is Boltzmann’s constant, p*K*_a_^0^ is the intrinsic acid dissociation constant of the amino acid model compound, and Δ*u*_elec_ is the change in electrostatic energy. In addition to swap moves, configurational space in the *NVT* ensemble is sampled using atomic translations, chain pivot, and crankshaft moves at 300 K. Average protonation states are sampled over one million Monte Carlo sweeps where, in each sweep, *N* residue move attempts are carried out. Both a single monomer and a fibril (represented by ten planes, PDB: 2N0A) of α-synuclein are studied. The 2N0A structure was chosen as it includes all the 140 residues of α-synuclein. For the monomer, a single chain is simulated with all beads connected by harmonic springs. For the fibril, the middle part is kept as described above and connected with flexible N-terminal and C-terminal ends (see above and Figure 7a for a snapshot). All simulations were performed using the Faunus software,^47^ and an electronic notebook (Jupyter Notebook) for reproducing all simulation results is provided. This allows the reader to investigate alternative fibril structures, add a finite free monomer concentration, or explore a change in any model parameter. For more information, see https://doi.org/10.5281/zenodo.4729860.

## 3 Results

### 3.1 Formation of α-Synuclein Fibrils in Water

**Figure 2:**
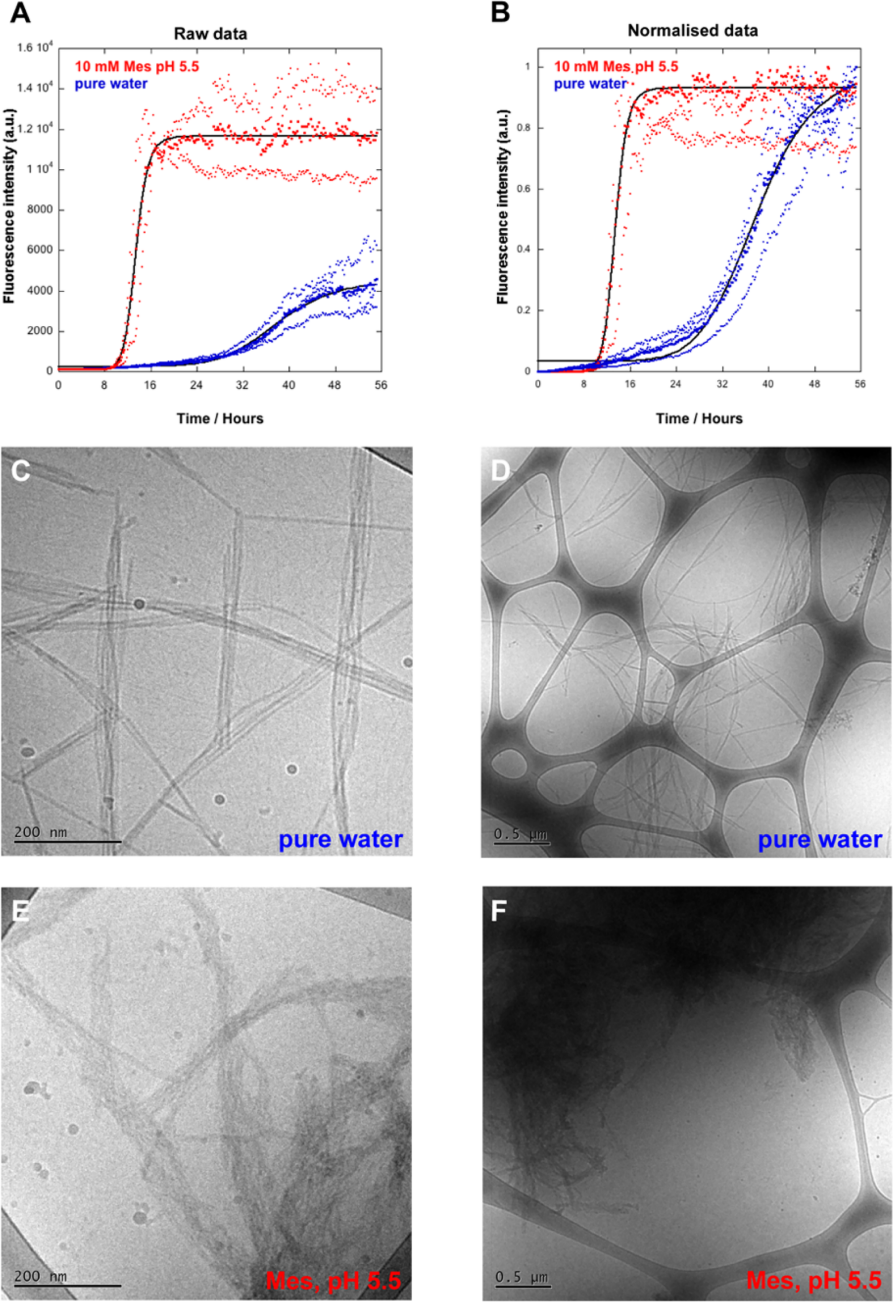
Ultrastructure of α-synuclein fibrils formed in pure water and 10 mM MES/NaOH, pH 5.5. Fibrils were formed by incubating the monomeric α-synuclein in a nontreated polystyrene plate at quiescent conditions. Samples were supplemented with 20 μM ThT and the fluorescence intensity measured at different time points. The protein concentration was 35 μM. (A) Raw data. Red: 10 mM MES/NaOH (pH 5.5), *t*_1/2_ = 13.4 h, two replicates. Blue: in pure water, *t*_1/2_ = 38.0 h, four replicates. The bold dots show the average of the replicates. Individual replicates are shown with thinner dots. The *t*_1/2_ was calculated by fitting a sigmoidal curve to the average data. (B) Normalized data, same method and data as those in A. Cryo-TEM images of the α-synuclein fibril samples collected after reaching the plateau in ThT fluorescence. (C and D) α-Synuclein fibrils formed in water. (E and F) α-Synuclein fibrils formed in 10 mM MES, pH 5.5.

The investigation of proton uptake by α-synuclein during fibril formation required that the reaction was followed in water, i.e. in the absence of buffer components except the protein. The kinetic behavior and the morphology of α-synuclein in water were therefore investigated and compared to fibrils formed in 10 mM MES/NaOH, pH 5.5. The fibril formation was followed by supplementing control samples with 20 μM ThT and monitoring the increase in ThT fluorescence (Figure 2). The pH measurements below were for protein only in water without ThT.

The aggregation kinetics (Figure 2a,b), indicate that amyloid fibrils form more slowly in pure water, with less steep transition and a *t*_1/2_ of 38.0 h, than in 10 mM MES/NaOH at pH 5.5 where *t*_1/2_ is 13.2 h.

The samples were harvested at the end of the aggregation reaction or after 56 h (Figure 2). Cryogenic transmission electron microscopy (Cryo-TEM) was used to evaluate if there were any ultrastructural differences between the fibrils formed in water and in 10 mM MES/NaOH pH 5.5. A clear difference is seen in the morphology of the fibrils formed in water and in buffer (Figure 2c–f). The fibrils formed in water are generally longer, thinner, and more dispersed over the grid than those formed in buffer. Some of the fibrils are very long, up to few micrometres. Short and thin fibrils are commonly close to the mature fibrils. The fibrils in water are more separated with longer distances between the fibrils and the filaments than the fibrils in the pH 5.5 buffer, which are more compact and tangled, forming big dense aggregates. This is presumably a result of stronger electrostatic repulsion between the protofilaments in water compared to buffer where salt screening becomes significant.

### 3.2 Detection of pH Increase during Fibril Formation by pH Electrode

**Figure 3:**
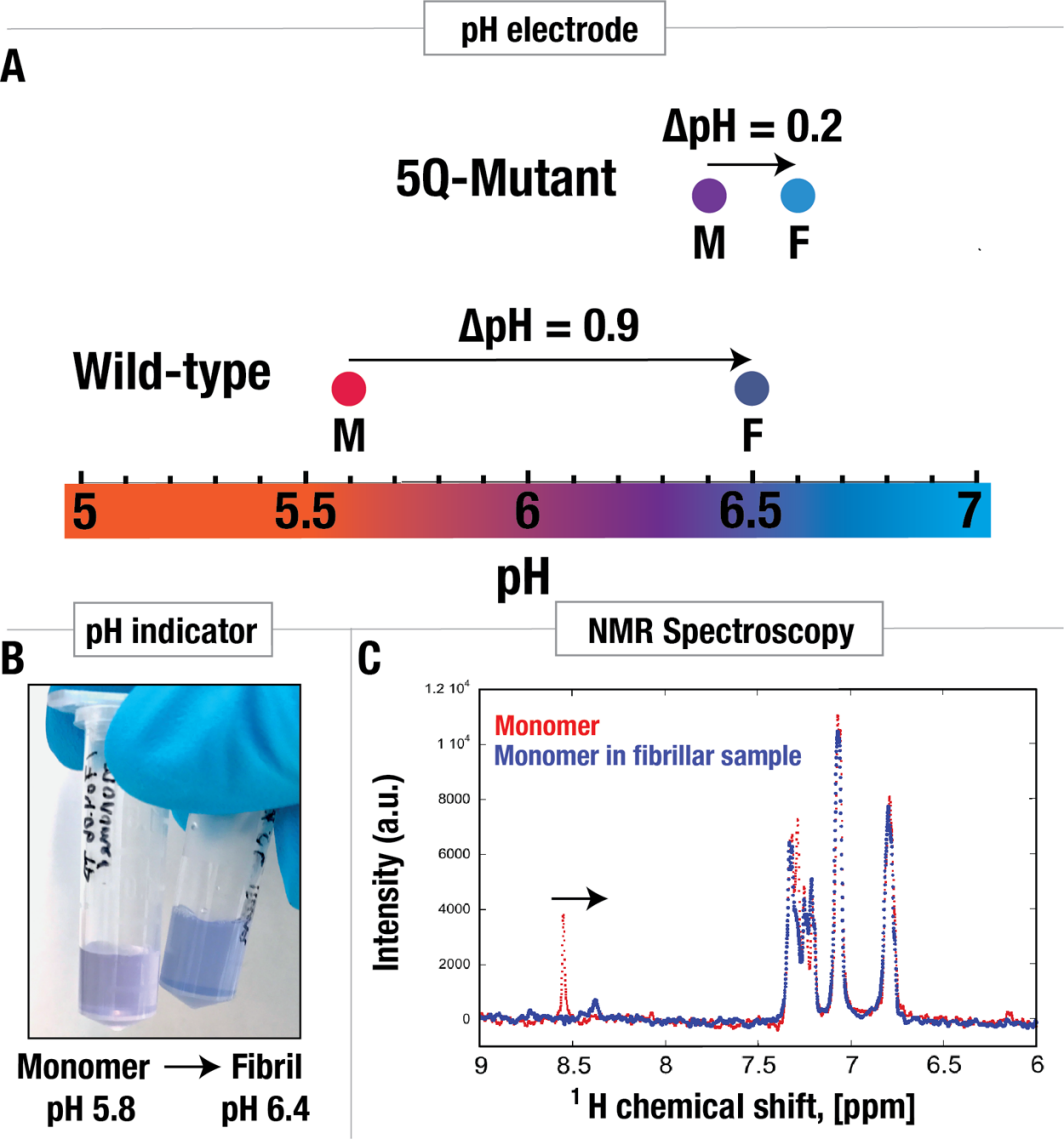
Change in pH during amyloid formation of wild-type α-synuclein and an α-synuclein 5Q mutant. (A) pH change during amyloid formation detected by pH electrode. The proteins (wild-type α-synuclein and the 5Q mutant) were isolated in pure water, and pH was measured before and after fibril formation. On average, pH changed from pH 5.6 to pH 6.5 for the wild-type protein and on average from pH 6.4 to 6.6 for the 5Q mutants. (B) pH change detected using the pH sensitive dye, resazurin. Monomeric α-synuclein in weak buffer containing resazurin, at pH 5.8, resulted in purple color. The fibrillar sample gave rise to blue color. For comparison, the pH was measured to be 6.4 using a pH electrode. (C) pH change detected using NMR spectroscopy using the δ2 proton of histidine in monomeric α-synuclein as a pH sensor. The red and blue traces show the spectrum before and after fibril formation, respectively. The large decrease in chemical shift detected for the monomer in the fibrillar sample is indicative of a pH increase.

Using pure water as a solvent makes it possible to measure the effect of amyloid fibril formation on the pH of the sample and subsequently use the pH to calculate the average shift in p*K*_a_ values. In water without buffer, any changes in pH of the sample depends only on changes in p*K*_a_ values that occur upon aggregation. Alteration of the p*K*_a_ values will change the number of free protons in solution, i.e. the pH of the sample. Therefore, a recorded change in pH within the sample (closed system) can be related to changes in p*K*_a_ values during fibril formation. The pH of the sample was measured using a pH electrode before and after the formation of amyloid fibrils. An increase of as much as 0.9 pH units (from pH 5.6 to 6.5) was detected for 20 μM α-synuclein in water (Figure 3a). From this pH change, it is possible to calculate the change in the apparent p*K*_a_^ave^ values of α-synuclein during fibril formation (see below). The effect on pH is amplified at higher protein concentration (70 μM) (Table S1).

To investigate if the pH increase is related to the high density of acidic residues in the C-terminal tail, the experiment was repeated for a mutant in which five acidic (glutamic acid) residues in the C-terminal tail had been replaced with noncharged polar (glutamine) residues (see Figure 1), denoted the 5Q mutant. The increase in pH during fibrillation was only 0.2 pH units for the 5Q mutant (from pH 6.4 to 6.6; Figure 3a) at 20 μM and slightly higher at 70 μM (Table S1).

**Figure 4:**
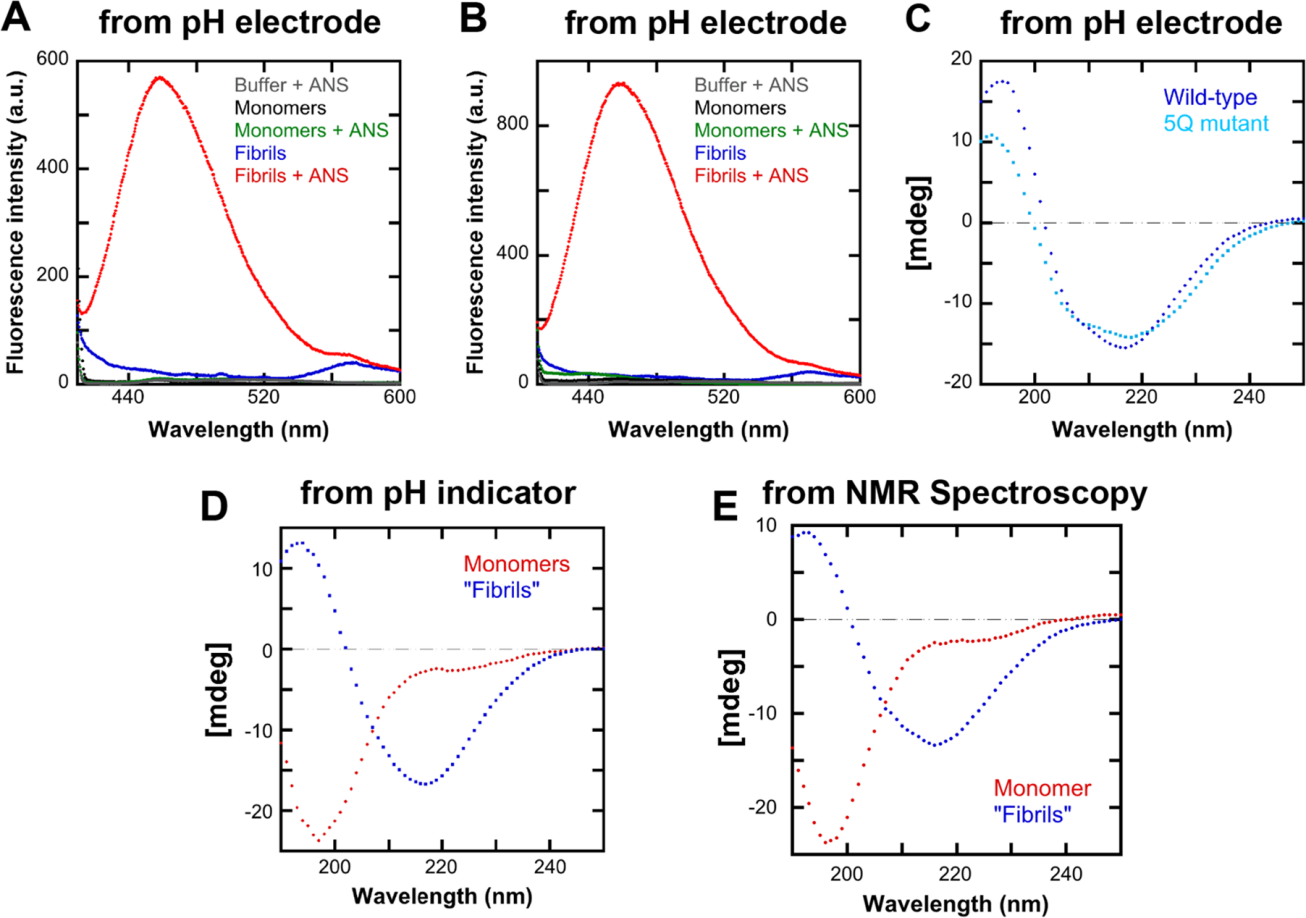
Verification of the presence of fibrils by optical spectroscopy. (A) ANS Fluorescence spectra before and after fibril formation of wild-type α-synuclein in water with the pH change measured using a pH electrode. (B) ANS Fluorescence spectra before and after fibril formation of the α-synuclein 5Q mutant in water with the pH change measured using a pH electrode. (C) CD-spectra before and after fibril formation of wild-type and 5Q mutant α-synuclein in water with pH changes measured using a pH electrode. (D) CD-spectra of wild-type α-synuclein before and after fibril formation in a weak buffer containing resazurin. (E) CD-spectra of α-synuclein in 100% D_2_O before and after fibril formation as monitored using NMR spectroscopy.

The secondary structure of the protein was studied using far-UV CD spectroscopy before and after fibril formation to verify the conversion of monomeric protein (mostly random coil structure) to amyloid fibrils (mostly β-sheet structure) (Figure 4c). As can be seen from Figure 4c, the fibrillar sample gave a CD spectrum that had a negative peak at 217 nm, a typical characteristic of a sample containing β-sheet structure, which indicates the conversion of the monomers to fibrils. Aliquots of the same samples were also supplemented with the fluorescent dye, ANS, and the fluorescence spectra were recorded. The fluorescence intensity of the fibrillar sample increased significantly compared to monomers (Figure 4a and 4b). This confirms the presence of amyloid fibrils in the fibrillar sample.

### 3.3 Change in pH Detected with pH Indicator

The pH change during fibril formation was also monitored using a complementary method that does not involve a pH electrode. α-Synuclein was allowed to form fibrils in a weak buffer system, constituting 0.5 mM MES and 12.5 μM resazurin, 0.02% NaN_3_ at pH 5.8. Resazurin is a pH indicator that shows a color change from purple to blue from pH 5.5 to 6.5. The starting pH of the sample was pH 5.8, giving the sample a purple color. The sample was then allowed to fibrillate, and the color of the fibril containing sample was compared to the monomeric sample (Figure 3b).

A change in the color from purple to blue was observed as fibrils were formed, indicating an increase in pH. For comparison, the pH of this sample was also measured to 6.4 with a pH electrode after fibril formation, in accordance with the color change. As before, CD-spectra were recorded before and after the fibril formation, to verify the conversion from monomeric to fibrillar sample (Figure 4d). Absorbance spectra were also recorded for comparison between the monomeric and fibrillar samples with indicator, but could not be used for quantification of the pH shift because of extensive baseline elevation for the fibrillar sample due to light scattering. Therefore, the indicator data serve as a qualitative confirmation of the upshift in pH.

### 3.4 Increase in pH Detected with NMR Spectroscopy

In addition to using a pH sensitive dye, solution NMR spectroscopy was used as a second complementary method to report on the change in pH during fibril formation. Here, the chemical shift of H^δ2^ of the imidazole ring in His50 of the monomer was used to monitor the pH.^48−50^ The p*K*_a_ value of histidine within the monomeric α-synuclein in 20 mM sodium phosphate buffer, pH 7, had previously been measured to be 6.78.^30^ At p*K*_a_ ± 1, the buffer capacity of the imidazole side chain is within 33% of its maximum, and at p*K*_a_ ± 1.5, it is within 10% of its maximum. The pH of monomeric and fibrillar samples in water (pH 5.6 to 6.5) falls within the buffer region of the histidine residue, making it possible to utilize it as a pH sensor. All the signals obtained in this experiment come from monomers, as the fibrils are too big to be observed by liquid state NMR (see in more detail in next paragraph), and therefore we can assume that any change in protonation of His50 is due to the change in pH of the solution and not due to p*K*_a_ perturbations resulting from conformation changes. A sample containing 70 μM α-synuclein in 99.9% D_2_O was analyzed by solution state 1D ^1^H (proton) NMR before and after amyloid fibril formation. The peak corresponding to the H^δ2^ of the imidazole ring in His50 shifted from 8.55 to 8.38 ppm (Figure 3c). CD-spectra were recorded before and after the fibril formation, to verify the formation of fibrils (Figure 4e).

### 3.5 2D Heteronuclear and Diffusion Ordered NMR Spectroscopy

**Figure 5:**
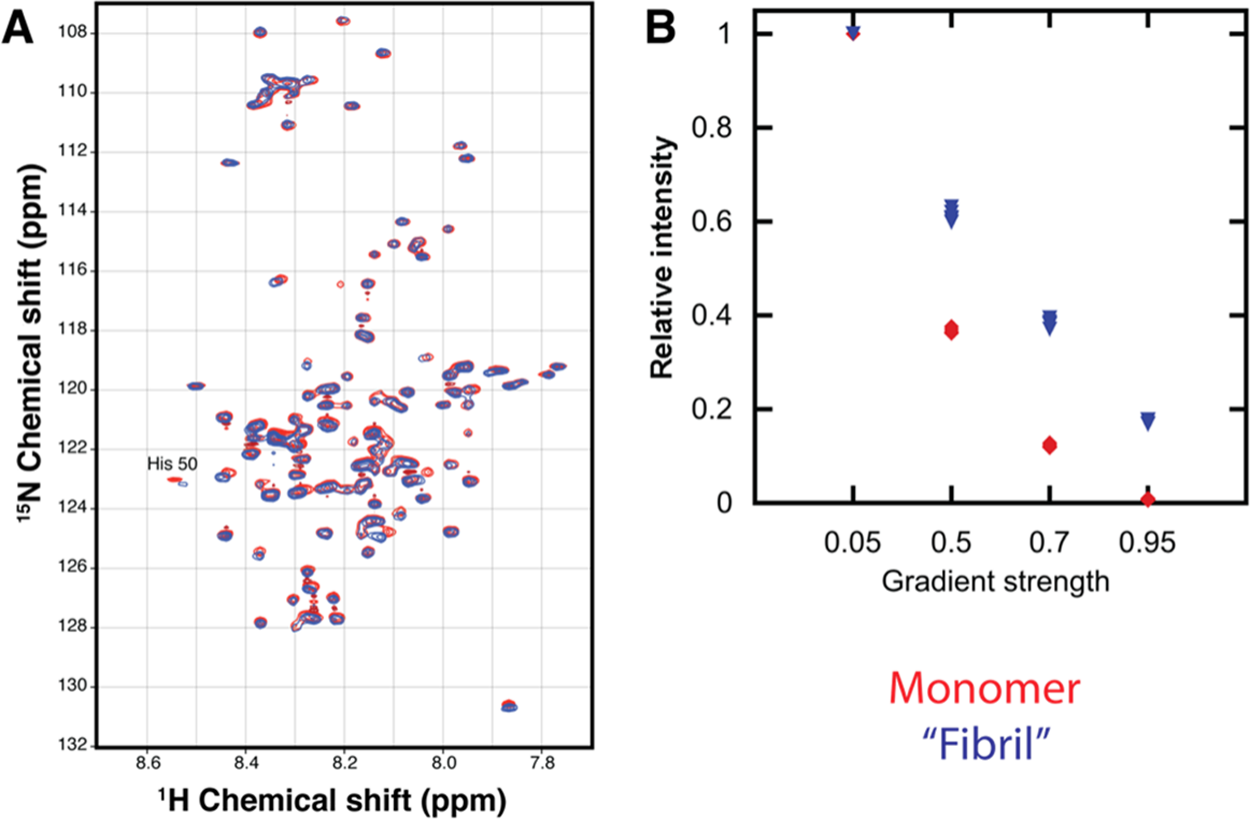
(A) 2D ^15^N-^1^H HSQC NMR spectra of ^15^N labeled α-synuclein in 5% D_2_O/95% H_2_O. (B) Relative intensities from diffusion ordered (DOSY) NMR spectra. Monomer signals in the absence of fibril are shown in red (diamonds in B), and in the presence of fibrils in blue (triangles in B). In B, the signal intensity is calculated relative to the intensity at a gradient strength of 0.05 of max strength.

2D ^15^N–^1^H HSQC NMR spectra showing resolved signals from amides in nearly all amino acid residues were run on both the monomeric and fibrillar sample (Figure 5a). The chemical shift differences between the two samples were very limited except for the region around His50.

Diffusion ordered NMR spectroscopy (DOSY) was used to distinguish between signals originating from monomers (faster diffusing species) and fibrils (slower diffusing species). The highest gradient strength used was strong enough to suppress the signals in the purely monomeric sample (Figure 5b). The signals in the fibril containing sample were only slightly less suppressed, indicating that they originate from monomeric species in fast exchange with a slower diffusing species such as fibrils rather than from the fibril itself. We can say that chemical shift change in the His50 originates from the unstructured monomers in both the monomeric and the fibrillar sample and is presumably due to a change in pH, causing a change in the protonation state of the His50.

### 3.6 Continuous Measurement of pH during Fibril Formation

**Figure 6:**
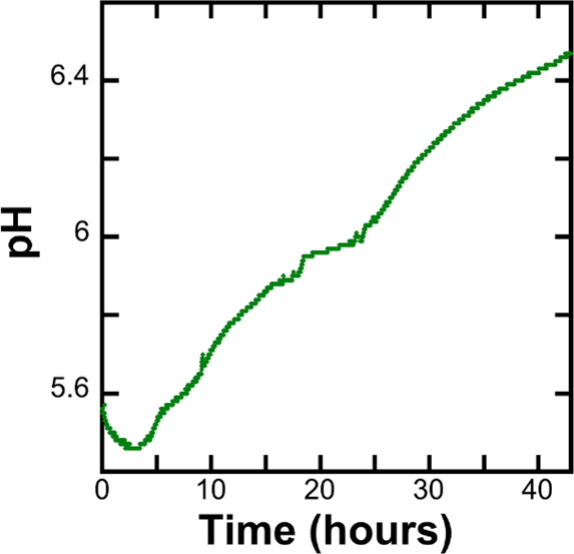
Time evolution of pH followed with pH electrode using Probe Drum. 70 μM sample was stirred at 237 rpm at 37 °C.

The pH of a solution containing freshly purified monomeric α-synuclein was measured continuously during formation of amyloid fibrils using a Probe Drum instrument equipped with a pH electrode. A small pH drop was detected at the start of the aggregation (first 3 h), followed by a gradual increase in pH over more than 40 h (Figure 6). Other samples, supplemented by ThT, allowed simultaneous measurement in the same sample of pH and ThT fluorescence as well as light scattering. These data show that the onset of the pH increase coincided with the onset of ThT fluorescence or scattering (Figure S1).

### 3.7 Calculations of the Average p*K*_a_ Upshift

As mentioned above, an increase in pH from 5.6 to 6.5 was observed for 20 μM α-synuclein in water. The experiments were performed in a closed system with a defined number of protons which makes it possible to calculate the change in the average apparent p*K*_a_ value (Δp*K*_a_^ave^) from the change in pH during the fibril formation (ΔpH). In order to do so the degree of protonation, *Q*, was calculated for each individual titratable acidic group in α-synuclein (including the C-terminus), according to
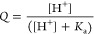
This was done by assuming that the p*K*_a_ values, and therefore the dissociation constants (*K*_a_) of the acidic groups of the monomeric α-synuclein, were the same as published by Croke et al.^30^ The average protonation state of the acidic groups *Q*^ave^ was calculated from the protonation state, *Q*, of individual groups.

By knowing the number of titratable acidic groups (# acidic groups) at this pH range, the *Q*^ave^, and the concentration (*C*) of protein molecules, the number of protons bound to the acidic groups can be calculated according to
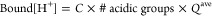
The number of protons free in solution (free[*H*^+^ ]_mono_) in the monomeric sample was then calculated from the pH value of the sample:
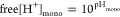
The total number of protons in the monomeric sample (total[H^+^]_mono_) is equal to the sum of the bound and the free protons:
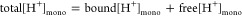
The total proton concentration in the monomeric sample (total[H^+^]_mono_) and the fibrillar sample (total[H^+^]_fib_) was assumed to be the same, as the pH change occurs within the same sample, a closed system:
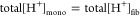
From now on we will consider the fibrillar sample. The concentration of protons free in solution in the fibrillar sample (free[H^+^]_fib_) was calculated from the pH measured in the fibrillar sample:
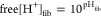
The total concentration of protons bound to the acidic groups (bound[*H*^+^]_fib_^tot^) within the fibrillar sample was then calculated according to
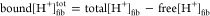
We have observed that, during the aggregation of α-synuclein, the monomers are not fully consumed into fibrils, in agreement with Gray et al. (2015).^51^ Therefore, the remaining monomer concentration (*C*_fib_^mono^) after fibril formation in water was measured (see SI, Figures S2 to S4)) and was taken into account in the calculations. The monomeric *Q*^ave^ was assumed to be the same in both samples. The monomeric *Q*^ave^ and the monomeric concentration in the fibrillar sample (*C*_fib_^mono^) was used to calculate the number of protons bound to monomers in the fibrillar sample (bound[*H*^+^]_fib_^mono^).
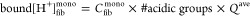
From that the number of protons bound to fibrils within the fibrillar sample was calculated (bound[H^+^]_fib_^fib^*)*
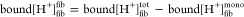
To calculate the average *K*_a_ for the fibrillar sample, the concentration of acidic protein side-chain groups in the fibrils that were not protonated (free[*P*]_fib_^fib^*)* was calculated according to
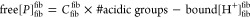
From that the proton equilibrium constant *K*_a_ for the fibrillar sample could be obtained:
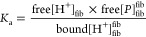
The *K*_a_ was then used to get the apparent p*K*_a_^ave^ value of the acidic groups within the fibrillar sample:
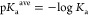
For comparison, the apparent p*K*_a_^ave^ was also calculated for the 5Q mutant. Here, *Q*^ave^ was calculated using the p*K*_a_ value of the remaining acidic residues, i.e. five groups less than for the wild-type protein. The concentration of free protons in solution was then calculated from the measured pH values of the 5Q mutant sample, before and after fibril formation.

The calculated apparent p*K*_a_^ave^ values of the fibrillar sample and the Δp*K*_a_^ave^ are given in Table 2. The Δp*K*_a_^ave^ for the wild-type sample was 1.1 while for the 5Q mutant, having 5 less acidic groups in the tail, it was 0.5. This suggests that this increase in p*K*_a_ values during amyloid formation of α-synuclein is related to the high density of acidic residues in the C-terminal tail.

**Table 2:** Apparent p*K*_a_^ave^ Values of the Acidic Residues of α-Synuclein Calculated for Both the Monomers and Fibrils^a^

	p*K*_a_ monomers	p*K*_a_ fibrils	Δp*K*_a_^ave^
Wild-type	4.3	5.4	1.1
5Q mutant	4.2	4.7	0.5

a Based on this, the upshift in the apparent p*K*_a_^ave^ values, Δp*K*_a_^ave^, was calculated.

The contribution of autoionization of water was calculated and was found to have no pronounced effect on the calculated p*K*_a_^ave^, with a 0.0004 units higher Δp*K*_a_^ave^ value for the wild-type α-synuclein, i.e. beyond the precision of our measurements.

The number of protons taken up per monomer during fibril formation were calculated to be about 2 protons per monomer in nonbuffered closed system with a defined number of protons (our experimental system), which partially neutralized the protein.

### 3.8 Molecular Modeling of Protonation States

#### 3.8.1 α-Synuclein Fibril

**Figure 7:**
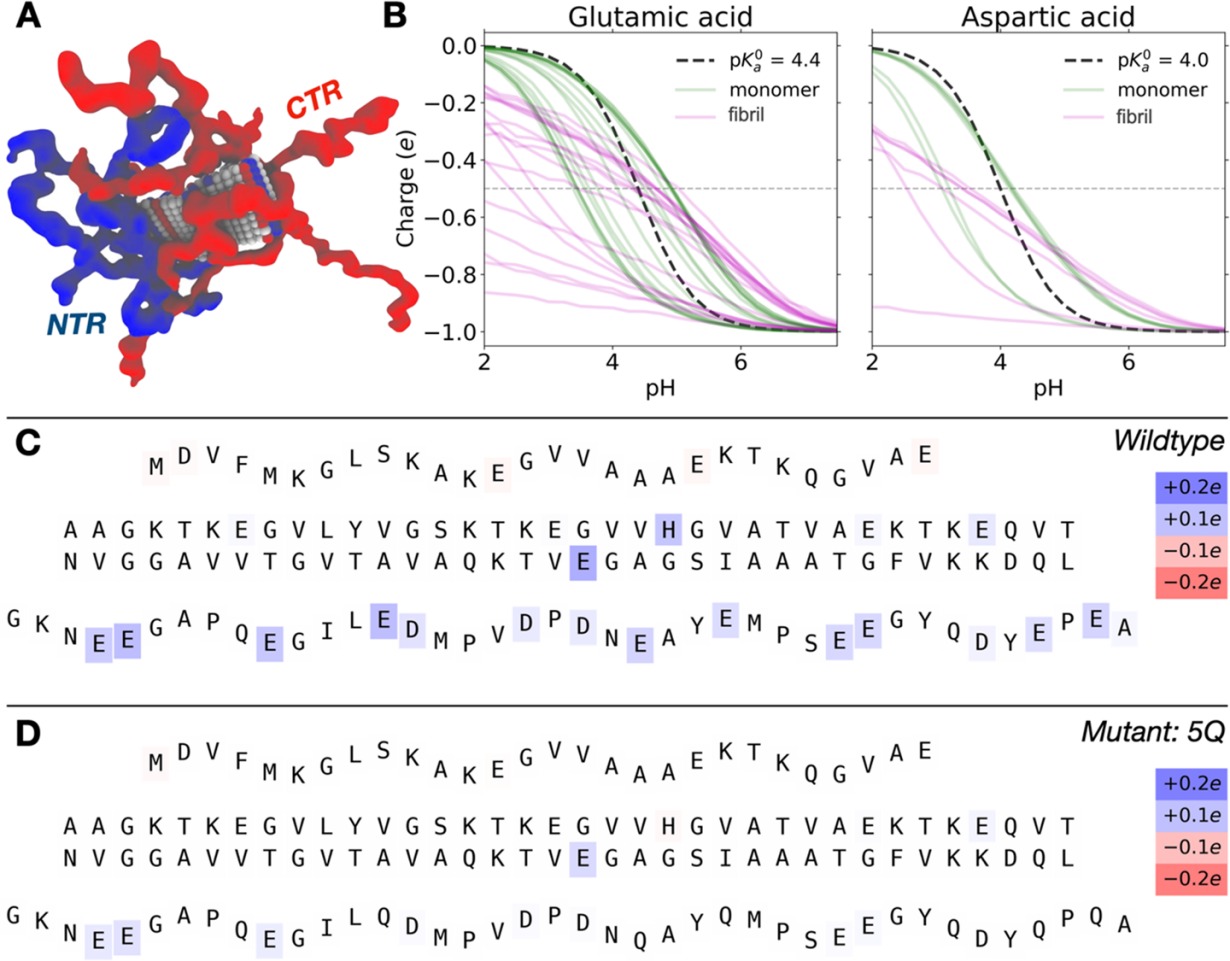
Results from Monte Carlo simulations of α-synuclein monomer and fibril (10 planes of a fibril as found in PDB: 2N0A) states. (A) Snapshot showing the rigid middle part of the fibril, flanked by flexible N-termini (blue) and C-termini (red). (B) Partial charge of carboxyl groups of glutamic acid and aspartic acid in the fibril (magenta) and in the monomer (green) as a function of pH. The dashed, black line shows the nonperturbed titration curve given by the model p*K*_a_^0^ value. (C) Differences in residue partial charges between α-synuclein in a monomer and in a fibril at constant pH 6.5. Shifts (charge in fibrillar minus charge in monomeric state) are indicated by the colorbars and letters correspond to the amino acid sequence with a rigid middle part surrounded by flexible (shown in “wavy” text) N-terminal and C-terminal parts. (D) Same as (C), but for the 5Q-mutant.

The ionization states of α-synuclein monomer and fibril (represented by ten planes, PDB: 2N0A) were studied using constant pH Metropolis Monte Carlo simulations; see Figure 7. Differences in residue partial charges in a monomeric and fibril state were calculated for both the wild-type α-synuclein and the α-synuclein 5Q mutant at constant pH 6.5 (Figure 7) and constant pH 5.5 (Figure S5).

#### 3.8.2 α-Synuclein Monomers

**Figure 8:**
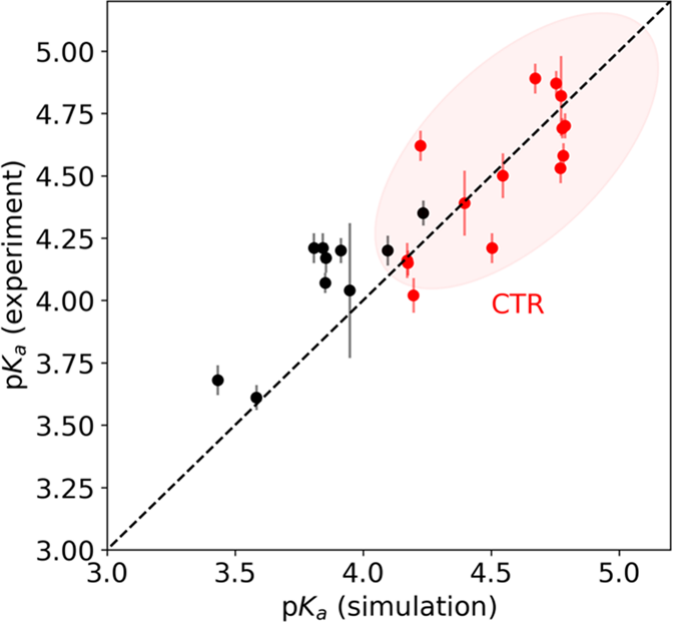
Correlation between measured^30^ and simulated stoichiometric p*K*_a_-values of aspartates and glutamates in α-synuclein monomers. The Metropolis Monte Carlo simulations were performed at 15 mM salt and the p*K*_a_-value was defined as the pH where the degree of ionization is 0.5. Values marked in red indicate that the sites are found in the C-terminal tail. Only a single peptide chain is present in the simulation cell, corresponding to infinite protein dilution, while the ionic strength was set to 15 mM.

In addition to experiments, the ionization state of α-synuclein monomer can be estimated using Metropolis Monte Carlo Computer simulations. To validate the peptide interaction model described earlier, we first studied the stoichiometric p*K*_a_-values of aspartate and glutamate side chains in an isolated α-synuclein monomer. These have previously been measured using NMR,^30^ and in Figure 8 we show how the experiment and simulation correlate.

## 4 Discussion

### 4.1 Proton Uptake during Amyloid Fibril Formation

In this work, the effect of aggregation on the p*K*_a_ values of α-synuclein was investigated and an increase in p*K*_a_ was observed by measuring the change in pH during fibril formation of α-synuclein in water, using three complementary methods. A pH increase from 5.6 to 6.5 for 20 μM α-synuclein was quantified using a pH electrode. This implies a proton uptake, i.e. an increased affinity for protons, indicating an upshift in the apparent p*K*_a_ values during fibril formation. Based on those results, the change in the apparent average p*K*_a_ value (Δp*K*_a_^ave^) of the titratable acidic residues in α-synuclein was calculated to be Δp*K*_a_^ave^ = 1.1, i.e. an increase in average p*K*_a_ values from 4.3 to 5.4.

The proton uptake during fibril formation in a nonbuffered closed system was found to be about 2 protons per monomer, in good agreement with the simulations performed at pH 6.5, which also showed an uptake of about 2 protons per monomer during fibril formation.

In a closed system, the protons taken up by the fibril must come from somewhere. This can only in part be explained by the reduction in proton concentration in the water, and indeed the protein itself serves as an internal buffer. Protons released by the monomeric fraction are taken up by the fibrillar fraction. As fibril formation proceeds, the monomers that remain in solution (having lower apparent p*K*_a_^ave^ values compared to fibrils) release protons, which are in turn taken up by the monomers that become a part of a fibril (having higher apparent p*K*_a_^ave^ values of the acidic residues). Therefore, the charge state of α-synuclein in the monomeric state and fibrillar state differ in the fibrillar sample. After fibril formation, the net charge of free monomers has increased while the net charge of monomers in the fibrils have decreased. This lowers the electrostatic repulsion within the fibril.

It should be noted that the monomeric p*K*_a_ values used in all the calculations are obtained from Croke et al. for 250 μM α-synuclein in buffer.^30^ However, buffer and protein concentrations affect the ion binding affinities of charged proteins due to electrostatic salt- and self-screening.^52,53^ The protein concentration in our samples, 20 μM, was more than 10 times lower than in the case of Croke et al., and the samples contained no other buffer than the protein itself. Also, from our studies, we see that the pH value of pure monomer in water is 5.6, indicating that the monomeric p*K*_a_ values might be closer to this value than to 4.3.^30^ In order to account for this uncertainty, the p*K*_a_ upshift was thus recalculated using several values for the p*K*_a_ values of monomer in the absence of fibrils. The result showed that the average upshift in p*K*_a_ value would not be much different if the p*K*_a_ values of the monomers would be higher (see SI, Figure S6).

### 4.2 Result of High Density of Acidic Residues within the Unstructured C-Terminal Tail?

The pH change during fibril formation was measured in the same way for an α-synuclein 5Q mutant containing five fewer acidic residues within the C-terminal tail, making the tail less acidic and less charged. This was done in order to gain better insight into the effect of the high charge density in the C-terminal tail, to investigate if the upshift in p*K*_a_^ave^ could be explained by the close proximity of acidic residues of the unstructured C-terminal tails extending from the fibril core. The pH increase during fibrillation was significantly lower for the 5Q mutant, giving an average pH increase from 6.4 to 6.6 for a sample of 20 μM α-synuclein. The p*K*_a_^ave^ was calculated to increase from 4.2 to 4.7, or a Δp*K*_a_^ave^ of 0.5 was found. These results demonstrate that decreased acidity and charge density of the C-terminal tail results in a smaller pH increase during fibril formation. This implies that the pH increases during fibril formation (proton uptake), and therefore, the upshift of p*K*_a_ values during amyloid formation of α-synuclein is linked to the high density of the acidic residues in the C-terminal tail. Those results were in good agreement with the results obtained from Monte Carlo simulations, also showing significantly less effect on protonation states during fibril formation of the 5Q mutant.

### 4.3 Electrostatically induced p*K*_a_ Shifts

Our results are line with a previous study by Urry et al. (1993) on upshifted p*K*_a_ values. They demonstrated a correlation between the decrease in density of acidic residues and the p*K*_a_ decrease, and suggested that the p*K*_a_ upshifts are primarily electrostatically induced and not induced due to desolvation.^11^ Upshifts in p*K*_a_ values due to the high density of acidic residues on protein surfaces of halophilic proteins has been reported.^54^ Likewise, Kesvatera et al. found an asymmetry in p*K*_a_ values in the EF-hand protein S100G with highly upshifted p*K*_a_ values in the highly acidic loop regions and unperturbed or down-shifted values at the opposite end of the protein.^22^ These observations are in accordance with our system where the extent of the upshifts was related to the charge density within the tails. After submission of this work, a study appeared with chemical shift changes in the C-terminal tail of α-synuclein upon fibril formation measured by solid-state NMR indicating a similar upshift in p*K*_a_ values.^55^

Upshifts in p*K*_a_ values due to changes in charge–charge interactions can be understood through Coulomb law, by calculating the Coulomb force between two adjacent charges:
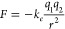
where *F* stands for the electrostatic force or the Coulomb force between two charges (*q*_1_, *q*_2_) separated by the distance *r*. When C-terminal tails come together at the fibrillar surface, the distance between the charges of different tails decreases, increasing the Coulomb force between tails. The increase in electrostatic force between charges is counteracted by upshift in p*K*_a_ values, making the tails proportionally more neutral (lower *q*_1_ and *q*_2_) and therefore decreasing the net force between adjacent tails.

### 4.4 Molecular Simulations

Figure 8 shows that the simulated results for monomeric α-synuclein are in fair agreement with NMR experiments,^30^ with similar upshifts and downshifts for aspartic and glutamic acids; the encircled red points correspond to residues in the C-terminal region. While deviations are expected due to the simplified interaction scheme, the data show that general trends in the observed p*K*_a_ shifts can be explained by an approximate electric potential due to the surrounding chemical environment.

Simulation data are in agreement with experimental data, and both show that protons are taken up during fibril formation of α-synuclein. Figure 7a shows a simulation snapshot of an α-synuclein fibril where the rigid middle is surrounded by flexible N- and C-termini. Significant differences are observed between the protonation states of carboxyl groups in the monomeric state and the fibril (Figure 7b). In the α-synuclein monomer, the pH titration curves are approximately symmetric while highly stretched in the fibril where full protonation is never reached even at the lowest pH. Fibril formation thus leads to a significant change in proton binding capacitance. This is because the densely packed fibril is cationic at low pH (see Figure S7), and the carboxyl groups are thus exposed to a strong *positive* potential, opposing protonation. The opposite is seen at high pH where the electrostatic environment is *negative* and protonation is favored compared to ideal titration. Figure 7b highlights the difficulty in describing electric perturbations by a simple upshift or downshift of the thermodynamic p*K*_a_-value. This is formally seen in the definition of the stoichiometric or apparent acid dissociation constant, *K*_a_^*^ = *K*_a_Γ(pH), where Γ is an activity coefficient product reflecting the local chemical environment of the site. Since Γ in nontrivial ways varies with pH, conformation, and other solution conditions, so does *K*_a_^*^. Thus, the p*K*_a_ “shift” is in reality a function of pH whereby the resulting titration curve can deviate far from a merely translated Henderson–Hasselbalch behavior. Still, from pH 6 and up, the titratable sites in the C-terminal region are on average predicted to be more protonated in the fibrillar state than in the monomeric state which of course agrees with the qualitative notion of a p*K*_a_ upshift during fibrillation.

It is instructive to trace the changes in the average protonation state of individual residues when embedding a monomer into a fibril. This is shown in Figure 7c where the FASTA letter background color shows the charge increment (blue) or decrement (red). At pH 6.5 the largest differences are observed in the C-terminal with all carboxyl groups being less negatively charged than in the monomer. This is due to the strong negative electric potential exerted by the surrounding C-terminal ends, causing proton uptake. In agreement with our experimental observations, the charge shifts are strongly subdued in the 5Q mutant (Figure 7d) and only marginal charge shifts between α-synuclein monomers and fibrils are observed. It is important to note that the simulations are performed at constant pH, i.e. fully buffered, while in the experiments proton uptake upon fibril formation is monitored through a pH change.

It has been argued that an elimination of transient interactions^32,56^ between the N- and C-terminus of wild-type monomers upon formation of fibrils could be an alternative explanation to the p*K*_a_ upshift. However, the results presented in Figure 7c and 7d show asymmetric perturbations with very small changes in the N-terminus. It should be emphasized that all residues are included in the simulations and we do not add restraints to the monomer conformation; it is thus allowed to take any transient conformation governed by the sequence. We see a clear change in the charge states in the C-terminus when monomer forms fibrils, and that the effect is strongly subdued for the 5Q mutant (see Figure 7c and d). The combination of our experimental and theoretical work strongly supports that the close stacking of the C-termini has a major role in the p*K*_a_ upshift during fibril formation.

### 4.5 Driving Force of Fibril Formation

The thermodynamic stability of proteins is dependent on the ionization state of the different amino acid residues. As seen from Coulomb’s law, the p*K*_a_ values of the residues within α-synuclein, and therefore the charge density of the C-terminal tail, can affect the thermodynamic stability of the fibrils. A decrease in electrostatic repulsion between adjacent tails, due to the p*K*_*a*_ upshifts, results in the fibril formation being thermodynamically more favorable.

According to literature, the hydrophobic effect and hydrogen bonding are two main intermolecular interactions stabilizing amyloid fibrils.^37,57−60^ Similar to formation of globular protein structures, the fibril core is stabilized through the hydrophobic effect and hydrogen bonding within the β-sheets of the amyloid structure,^57^ although hydrogen bonding may be more or less iso-energetic over monomer and fibril as long as all potential hydrogen bonding groups are engaged in hydrogen bonds with water molecules or other protein groups. This is reflected in the known amyloid structures, where the fibrillar core has been found to profoundly constitute hydrophobic and polar, noncharged residues (Figure 1).^31,61,62^ Highly charged C-terminal tails and extensive electrostatic repulsion between adjacent monomers will presumably oppose the hydrophobic effect, making fibril formation less favorable. Without upshifts in the p*K*_a_ values and a higher degree of neutralization of the tails on the fibrillar surface, the favorable interactions between adjacent monomers would presumably be overridden by the unfavorable electrostatic repulsion.

### 4.6 Increase in p*K*_a_ Values and the Preventive Role of the Tail

Previous studies of α-synuclein have shown that truncation of the tail,^39,40,63,64^ charge-shielding,^40^ and neutralization^36,37^ affects the aggregation propensity. It has been suggested that the tail has a preventive role against aggregation.^39^ Protective characteristics of negative charges has been inferred for other systems; for example, the high number of negatively charged residues on the surface of halophilic proteins might prevent aggregation through electrostatic repulsion.^54^

It is interesting to speculate on the implications of the higher degree of neutralization of the tails within the fibrils, which makes the surface less charged at mildly acidic pH. The p*K*_a_ upshift and consequently the higher degree of neutralization presumably modulates the interaction between the fibrillar surface and the approaching monomers with implications for surface-catalyzed secondary nucleation.

### 4.7 Comparison to Other Systems

Perturbations of p*K*_a_ values are commonly found in enzyme active sites and within protein cores. Quantifications of p*K*_a_ upshifts are frequently reported in the literature. However, to our knowledge, the upshift in p*K*_a_ values during amyloid fibril formation has not been reported. Proton uptake or release during fibrillation has been observed for lysozyme and glucagon in a previous study.^65^ This leads to neutralization of the protein net charge and indicates an upshift in p*K*_a_ values within the fibrillar state in these cases.

In addition, p*K*_a_ perturbations have been observed during other self-assembly reactions. The p*K*_a_ value of a C-terminal carboxyl acid group was found to upshift during fiber and hydrogel formation of Fmoc-diphenylalanine.^66,67^ Additionally, upshifts in p*K*_a_ values of fatty acids were found to be a result of a decrease in intermolecular distance upon membrane self-assembly and dependent on the chain length, which modulates the intermolecular interactions and thereby the distance between the acidic headgroups at the interface.^68^ These additional examples demonstrate that p*K*_a_ value perturbations are likely to occur within all self-assembly processes of charged substances with ionizable groups to counteract the increase in local concentration of negative charges at surfaces upon a decrease in distance between molecules.

## 5 Conclusion

A significant upshift in p*K*_a_ values of at least 1.1 units on average accompanies amyloid fibril formation of α-synuclein. We demonstrated, measured, and calculated the upshift using three complementary experimental methods as well performing Metropolis Monte Carlo simulations on a comparable system. A significant change in proton binding capacitance upon fibril formation is due to the accumulation of titrating groups on the fibril surface. The significantly smaller upshift for an α-synuclein 5Q mutant having five less charged residues in the C-terminal tail implies that the magnitude of the upshift in p*K*_a_ is related to the density of acidic residues in the C-terminal tail. The observed p*K*_a_ perturbations during fibril formation provide further insights into the importance and the role of electrostatic interactions in the fibril formation of α-synuclein. The results have implications for other amyloid forming proteins and self-assembling systems in general, for which the methods reported here could provide quantitative information on p*K*_a_ perturbations and the role of electrostatic interaction in self-assembly processes.

## Supporting Information Available

The Supporting Information is available free of charge at https://pubs.acs.org/doi/10.1021/jacs.1c01925. Electronic Jupyter Notebooks for running and analyzing MC simulations are available at https://doi.org/10.5281/zenodo.4729860

Additional experimental sections: cloning of α-synuclein and α-synuclein 5Q mutant; the DNA sequence for wild-type α-synuclein; and the DNA sequence for 5Q α-synuclein mutant. Addition results: the effect of protein concentration on pH and Δp*K*_*a*_^ave^; fluctuations of average upshift in p*K*_a_ values during fibril formation due to increase of monomeric p*K*_a_ value; Metropolis Monte Carlo simulations of α-synuclein monomer and fibril at pH 5.5; protein Net-charge from Metropolis Monte Carlo Simulations; simultaneous measurements of the pH, ThT fluorescence and light scattering; and analysis of monomeric concentration remaining in solution after fibril formation in water (PDF)

## Abbreviations:

CD: circular dichroism
Cryo-TEM: Cryogenic transmission electron microscopy
DEAE: Diethylaminoethyl
DOSY: Diffusion ordered NMR spectroscopy
EDTA: ethylenediaminetetraacetic acid
FPLC: fast liquid protein chromatography
HSQC: heteronuclear single quantum coherence
IPTG: isopropyl thio-ß-D-galactoside
MC: Monte Carlo
MES: 2-(N-morpholino)ethanesulfonic acid
NMR: nuclear magnetic resonance
ON: overnight
PTFE: Polytetrafluoroethylene
SASA: solvent accessible surface area
SEC: size exclusion chromatography
ThT: thioflavin T

## References

[ref1] Tanford C. The Interpretation of Hydrogen Ion Titration Curves of Proteins. Adv. Protein Chem. 1963, 17, 69–165. 10.1016/S0065-3233(08)60052-2.

[ref2] Sörensen S. P. L.; Höyrup M.; Hempel J.; Paritzsch S. The capacity of egg-albumin to cimbine with acids or bases. Comptes rendus des Trav. du Lab. Carlsb. 1917, 12, 68–163.

[ref3] Zhou H. X.; Pang X. Electrostatic Interactions in Protein Structure, Folding, Binding, and Condensation. Chem. Rev. 2018, 118, 1691–1741. 10.1021/acs.chemrev.7b00305. 29319301 PMC5831536

[ref4] Braun-Sand S.; Warshel A. In Protein Folding Handbook, 2nd ed.; Buchner J., Kiefhaber T., Eds.; WILEY-VCH Verlag GmbH & Co. KGaA: Weinheim, 2008; Vol. 1, pp 163–200.

[ref5] Pace C. N.; Grimsley G. R.; Scholtz J. M. Protein Ionizable Groups: pK Values and Their Contribution to Protein Stability and Solubility. J. Biol. Chem. 2009, 284, 13285–13289. 10.1074/jbc.R800080200. 19164280 PMC2679426

[ref6] Perutz M. F. Electrostatic Effects in Proteins. Science (Washington, DC, U. S.) 1978, 201, 1187–1191. 10.1126/science.694508. 694508

[ref7] Yang A.-S.; Honig B. Electrostatic effects on protein stability. Curr. Opin. Struct. Biol. 1992, 2, 40–45. 10.1016/0959-440X(92)90174-6.

[ref8] Grimsley G. R.; Scholtz J. M.; Pace C. N. A summary of the measured pK values of the ionizable groups in folded proteins. Protein Sci. 2008, 18, 247–251. 10.1002/pro.19. PMC2708032 19177368

[ref9] Linderstrøm-Lang K. U. On the ionisation of proteins. Comptes rendus des Trav. du Lab. Carlsb. 1924, 15, 1–29.

[ref10] Schellman J. A.; Schellman C. G. Kaj Ulrik Linderstrøm-Lang (1896–1959). Protein Sci. 1997, 6, 1092–1100. 10.1002/pro.5560060516. 9144781 PMC2143695

[ref11] Urry D. W.; Peng S. Q.; Parker T. M.; Gowda D. C.; Harris R. D. Relative Significance of Electrostatic- and Hyfrophobic-Induced pKa Shifts in a Model Protein: The Aspartic Acid Residue. Angew. Chem., Int. Ed. Engl. 1993, 32, 1440–1442. 10.1002/anie.199314401.

[ref12] Tanford C.; Kirkwood J. G.; Tanford C. Theory of Protein Titration Curves. I. General Equations for Impenetrable Spheres. J. Am. Chem. Soc. 1957, 79, 5333–5339. 10.1021/ja01577a001.

[ref13] Buell A. K.; Hung P.; Salvatella X.; Welland M. E.; Dobson C. M.; Knowles T. P. J. Electrostatic Effects in Filamentous Protein Aggregation. Biophys. J. 2013, 104, 1116–1126. 10.1016/j.bpj.2013.01.031. 23473495 PMC3610013

[ref14] Thurlkill R. L.; Grimsley G. R.; Scholtz J. M.; Pace C. N. pK values of the ionizable groups of proteins. Protein Sci. 2006, 15, 1214–1218. 10.1110/ps.051840806. 16597822 PMC2242523

[ref15] Harris T. K.; Turner G. J. Structural Basis of Perturbed pKa Values of Catalytic Groups in Enzyme Active Sites. IUBMB Life 2002, 53, 85–98. 10.1080/15216540211468. 12049200

[ref16] Thomas P. G.; Russell A. J.; Fersht A. R. Tailoring the pH dependence of enzyme catalysis using protein engineering. Nature 1985, 318, 375–376. 10.1038/318375a0.

[ref17] Oda Y.; Yamazaki T.; Nagayama K.; Kanaya S.; Kuroda Y.; Nakamura H. Individual Ionization Constants of All the Carboxyl Groups in Ribonuclease HI from Escherichia coli Determined by NMR. Biochemistry 1994, 33, 5275–5284. 10.1021/bi00183a034. 7909691

[ref18] Petersson E. J.; Choi A.; Dahan D. S.; Lester H. A.; Dougherty D. A. A Perturbed pKa at the Binding Site of the Nicotinic Acetylcholine Receptor: Implications for Nicotine Binding. J. Am. Chem. Soc. 2002, 124, 12662–12663. 10.1021/ja028206i. 12392404

[ref19] Tanford C. The Interpretation Of Hydrogen Ion Titration Curves of Proteins. Adv. Protein Chem. 1963, 17, 69–165. 10.1016/S0065-3233(08)60052-2.

[ref20] Schwans J. P.; Sunden F.; Gonzalez A.; Tsai Y.; Herschlag D. Uncovering the Determinants of a Highly Perturbed Tyrosine pKa in the Active Site of Ketosteroid Isomerase. Biochemistry 2013, 52, 7840–7855. 10.1021/bi401083b. 24151972 PMC3890242

[ref21] Pahari S.; Sun L.; Alexov E. PKAD: a database of experimentally measured pKa values of ionizable groups in proteins. Database 2019, 2019, 1–7. 10.1093/database/baz024. PMC6389863 30805645

[ref22] Kesvatera T.; Jönsson B.; Thulin E.; Linse S. Focusing of the Electrostatic Potential at EF-hands of Calbindin D9k: Titration of Acidic Residues. Proteins: Struct., Funct., Genet. 2001, 45, 129–135. 10.1002/prot.1132. 11562942

[ref23] Laurents D. V.; Huyghues-Despointes B. M. P.; Bruix M.; Thurlkill R. L.; Schell D.; Newsom S.; Grimsley G. R.; Shaw K. L.; Treviño S.; Rico M.; Briggs J. M.; Antosiewicz J. M.; Scholtz J. M.; Pace C. N. Charge-Charge Interactions are Key Determinants of the pK Values of Ionizable Groups in Ribonuclease Sa (pI = 3.5) and a basic variant (pI = 10.2). J. Mol. Biol. 2003, 325, 1077–1092. 10.1016/S0022-2836(02)01273-1. 12527309

[ref24] Lindman S.; Xue W. F.; Szczepankiewicz O.; Bauer M. C.; Nilsson H.; Linse S. Salting the Charged Surface: pH and Salt Dependence of Protein G B1 Stability. Biophys. J. 2006, 90, 2911–2921. 10.1529/biophysj.105.071050. 16443658 PMC1414578

[ref25] Nick Pace C.; Alston R. W.; Shaw K. L. Charge-charge interactions influence the denatured state ensemble and contribute to protein stability. Protein Sci. 2000, 9, 1395–1398. 10.1110/ps.9.7.1395. 10933506 PMC2144688

[ref26] Elcock A. H. Realistic Modeling of the Denatured States of Proteins Allows Accurate Calculations of the pH Dependence of Protein Stability. J. Mol. Biol. 1999, 294, 1051–1062. 10.1006/jmbi.1999.3305. 10588906

[ref27] Shammas S. L.; Knowles T. P. J.; Baldwin A. J.; MacPhee C. E.; Welland M. E.; Dobson C. M.; Devlin G. L. Perturbation of the Stability of Amyloid Fibrils through Alteration of Electrostatic Interactions. Biophys. J. 2011, 100, 2783–2791. 10.1016/j.bpj.2011.04.039. 21641324 PMC3117150

[ref28] Breydo L.; Wu J. W.; Uversky V. N. α-Synuclein misfolding and Parkinson’s disease. Biochim. Biophys. Acta, Mol. Basis Dis. 2012, 1822, 261–285. 10.1016/j.bbadis.2011.10.002. 22024360

[ref29] Ulmer T. S.; Bax A.; Cole N. B.; Nussbaum R. L. Structure and Dynamics of Micelle-bound Human α-Synuclein. J. Biol. Chem. 2005, 280, 9595–9603. 10.1074/jbc.M411805200. 15615727

[ref30] Croke R. L.; Patil S. M.; Quevreaux J.; Kendall D. a.; Alexandrescu a. T. NMR determination of pKa values in α-synuclein. Protein Sci. 2011, 20, 256–269. 10.1002/pro.556. 21280118 PMC3048411

[ref31] Tuttle M. D.; Comellas G.; Nieuwkoop A. J.; Covell D. J.; Berthold D. A.; Kloepper K. D.; Courtney J. M.; Kim J. K.; Barclay A. M.; Kendall A.; Wan W.; Stubbs G.; Schwieters C. D.; Lee V. M. Y.; George J. M.; Rienstra C. M. Solid-state NMR structure of a pathogenic fibril of full-length human α-synuclein. Nat. Struct. Mol. Biol. 2016, 23, 409–415. 10.1038/nsmb.3194. 27018801 PMC5034296

[ref32] Wu K. P.; Weinstock D. S.; Narayanan C.; Levy R. M.; Baum J. Structural Reorganization of α-Synuclein at Low pH Observed by NMR and REMD Simulations. J. Mol. Biol. 2009, 391, 784–796. 10.1016/j.jmb.2009.06.063. 19576220 PMC2766395

[ref33] Sweers K. K. M.; van der Werf K. O.; Bennink M. L.; Subramaniam V. Atomic Force Microscopy under Controlled Conditions Reveals Structure of C-Terminal Region of α-Synuclein in Amyloid Fibrils. ACS Nano 2012, 6, 5952–5960. 10.1021/nn300863n. 22695112

[ref34] Del Mar C.; Greenbaum E. A.; Mayne L.; Englander S. W.; Woods V. L. Structure and properties of α-synuclein and other amyloids determined at the amino acid level. Proc. Natl. Acad. Sci. U. S. A. 2005, 102, 15477–15482. 10.1073/pnas.0507405102. 16223878 PMC1266128

[ref35] Der-Sarkissian A.; Jao C. C.; Chen J.; Langen R. Structural Organization of α-Synuclein Fibrils Studied by Site-Directed Spin Labeling. J. Biol. Chem. 2003, 278, 37530–37535. 10.1074/jbc.M305266200. 12815044

[ref36] McClendon S.; Rospigliosi C. C.; Eliezer D. Charge neutralization and collapse of the C-terminal tail of alpha-synuclein at low pH. Protein Sci. 2009, 18, 1531–1540. 10.1002/pro.149. 19475665 PMC2775220

[ref37] Buell A. K.; Galvagnion C.; Gaspar R.; Sparr E.; Vendruscolo M.; Knowles T. P. J.; Linse S.; Dobson C. M. Solution conditions determine the relative importance of nucleation and growth processes in α-synuclein aggregation. Proc. Natl. Acad. Sci. U. S. A. 2014, 111, 7671–7676. 10.1073/pnas.1315346111. 24817693 PMC4040554

[ref38] Gaspar R.; Meisl G.; Buell A. K.; Young L.; Kaminski C. F.; Knowles T. P. J.; Sparr E.; Linse S. Acceleration of α-synuclein aggregation. Amyloid 2017, 24, 20–21. 10.1080/13506129.2017.1292904. 28434341

[ref39] Levitan K.; Chereau D.; Cohen S. I. A.; Knowles T. P. J.; Dobson C. M.; Fink A. L.; Anderson J. P.; Goldstein J. M.; Millhauser G. L. Conserved C-terminal Charge Exerts a Profound Influence on the Aggregation Rate of α-synuclein. J. Mol. Biol. 2011, 411, 329–333. 10.1016/j.jmb.2011.05.046. 21689664 PMC3143250

[ref40] Hoyer W.; Cherny D.; Subramaniam V.; Jovin T. M. Impact of the Acidic C-Terminal Region Comprising Amino Acids 109–140 on α-Synuclein Aggregation in Vitro. Biochemistry 2004, 43, 16233–16242. 10.1021/bi048453u. 15610017

[ref41] Gaspar R.; Lund M.; Sparr E.; Linse S. Anomalous Salt Dependence Reveals an Interplay of Attractive and Repulsive Electrostatic Interactions in α-synuclein Fibril Formation. QRB Discovery 2020, 1, 1–11. 10.1017/qrd.2020.7. PMC10392692 37528959

[ref42] Nozaki Y.; Tanford C. [84] Examination of titration behavior. Methods Enzymol. 1967, 11, 715–734. 10.1016/S0076-6879(67)11088-4.

[ref43] Bundi A.; Wuthrich K. 1H-NMR Parameters of the Common Amino Acid Residues Measured in Aqueous Solutions of the Linear Tetrapeptides H-Gly-Gly-X-L-Ala-OH. Biopolymers 1979, 18, 285–297. 10.1002/bip.1979.360180206.

[ref44] Cantor C. R.; Schimmel P. R. Biophysical Chemistry: Part II: Techniques for the Study of Biological Structure and Function, 1st ed.; W. H. Freeman and Company: San Fransisco, 1980.

[ref45] Younan N. D.; Viles J. H. A Comparison of Three Fluorophores for the Detection of Amyloid Fibers and Prefibrillar Oligomeric Assemblies. ThT (Thioflavin T); ANS (1-Anilinonaphthalene-8-sulfonic Acid); and bisANS (4,4′-Dianilino-1,1′-binaphthyl-5,5′-disulfonic Acid). Biochemistry 2015, 54, 4297–4306. 10.1021/acs.biochem.5b00309. 26087242

[ref46] Wu D.; Chen A.; Johnson C. S. An Improved Diffusion-Ordered Spectroscopy Experiment Incorporating Bipolar-Gradient Pulses. J. Magn. Reson., Ser. A 1995, 115, 260–264. 10.1006/jmra.1995.1176.

[ref47] Stenqvist B.; Thuresson A.; Kurut A.; Vácha R.; Lund M. Faunus-A flexible framework for Monte Carlo simulation. Mol. Simul. 2013, 39, 1233–1239. 10.1080/08927022.2013.828207.

[ref48] Sachs D. H.; Schechter A. N.; Cohen J. S. Nuclear Magnetic Resonance Titration Curves of Histidine Ring Protons. I. Influence of Neighboring Charged Groups. J. Biol. Chem. 1971, 246, 6576–6580. 10.1016/S0021-9258(19)34152-3. 5132670

[ref49] Hass M. A. S.; Hansen D. F.; Christensen H. E. M.; Led J. J.; Kay L. E. Characterization of Conformational Exchange of a Histidine Side Chain: Protonation, Rotamerization, and Tautomerization of His61 in Plastocyanin from Anabaena variabilis. J. Am. Chem. Soc. 2008, 130, 8460–8470. 10.1021/ja801330h. 18540585

[ref50] Markley J. L. Observation of Histidine Residues in Proteins by Means of Nuclear Magnetic Resonance Spectroscopy. Acc. Chem. Res. 1975, 8, 70–80. 10.1021/ar50086a004.

[ref51] Marie G.; Dunning C. J.; Gaspar R.; Grey C.; Brundin P.; Sparr E.; Linse S. Acceleration of α-synuclein aggregation by exosomes. J. Biol. Chem. 2015, 290, 2969–2982. 10.1074/jbc.M114.585703. 25425650 PMC4317028

[ref52] Kesvatera T.; Jönsson B.; Thulin E.; Linse S. Measurement and Modelling of Sequence-specific pKa Values of Lysine Residues in Calbindin D9k. J. Mol. Biol. 1996, 259, 828–839. 10.1006/jmbi.1996.0361. 8683586

[ref53] Linse S.; Jönsson B.; Chazin W. J. The effect of protein concentration on ion binding. Proc. Natl. Acad. Sci. U. S. A. 1995, 92, 4748–4752. 10.1073/pnas.92.11.4748. 7761395 PMC41784

[ref54] Elcock A. H.; Mccammon J. A. Electrostatic Contributions to the Stability of Halophilic Proteins. J. Mol. Biol. 1998, 280, 731–748. 10.1006/jmbi.1998.1904. 9677300

[ref55] Kumari P.; Ghosh D.; Vanas A.; Fleischmann Y.; Wiegand T. Structural insights into α-synuclein monomer–fibril interactions. Proc. Natl. Acad. Sci. U. S. A. 2021, 118, e2012171118 10.1073/pnas.2012171118. 33649211 PMC7958257

[ref56] Bertoncini C. W.; Jung Y. S.; Fernandez C. O.; Hoyer W.; Griesinger C.; Jovin T. M.; Zweckstetter M. Release of long-range tertiary interactions potentiates aggregation of natively unstructured α-synuclein. Proc. Natl. Acad. Sci. U. S. A. 2005, 102, 1430–1435. 10.1073/pnas.0407146102. 15671169 PMC547830

[ref57] Fitzpatrick A. W.; Knowles T. P. J.; Waudby C. A.; Vendruscolo M.; Dobson C. M. Inversion of the Balance between Hydrophobic and Hydrogen Bonding Interactions in Protein Folding and Aggregation. PLoS Comput. Biol. 2011, 7, e1002169 10.1371/journal.pcbi.1002169. 22022239 PMC3192805

[ref58] Tsemekhman K.; Goldschmidt L.; Eisenberg D.; Baker D. Cooperative hydrogen bonding in amyloid formation. Protein Sci. 2007, 16, 761–764. 10.1110/ps.062609607. 17327394 PMC2203343

[ref59] Ma B.; Nussinov R. Simulations as analytical tools to understand protein aggregation and predict amyloid conformation. Curr. Opin. Chem. Biol. 2006, 10, 445–452. 10.1016/j.cbpa.2006.08.018. 16935548

[ref60] Buell A. K.; Dhulesia A.; White D. A.; Knowles T. P. J.; Dobson C. M.; Welland M. E. Detailed Analysis of the Energy Barriers for Amyloid Fibril Growth. Angew. Chem., Int. Ed. 2012, 51, 5247–5251. 10.1002/anie.201108040. 22489083

[ref61] Silvers R.; Colvin M. T.; Frederick K. K.; Jacavone A. C.; Lindquist S.; Linse S.; Griffin R. G. Aggregation and Fibril Structure of AβM01–42 and Aβ1–42. Biochemistry 2017, 56, 4850–4859. 10.1021/acs.biochem.7b00729. 28792214 PMC8254419

[ref62] Guerrero-Ferreira R.; Taylor N. M. I.; Arteni A. A.; Kumari P.; Mona D.; Ringler P.; Britschgi M.; Lauer M. E.; Makky A.; Verasdock J.; Riek R.; Melki R.; Meier B. H.; Böckmann A.; Bousset L.; Stahlberg H. Two new polymorphic structures of human full-length alpha-synuclein fibrils solved by cryo-electron microscopy. eLife 2019, 8, 1–24. 10.7554/eLife.48907. PMC6957273 31815671

[ref63] Van Der Wateren I. M.; Knowles T. P. J.; Buell A. K.; Dobson C. M.; Galvagnion C. C-terminal truncation of α-synuclein promotes amyloid fibril amplification at physiological pH. Chem. Sci. 2018, 9, 5506–5516. 10.1039/C8SC01109E. 30061982 PMC6048717

[ref64] Murray I. V. J.; Giasson B. I.; Quinn S. M.; Koppaka V.; Axelsen P. H.; Ischiropoulos H.; Trojanowski J. Q.; Lee V. M. Y. Role of α-Synuclein Carboxy-Terminus on Fibril Formation in Vitro. Biochemistry 2003, 42, 8530–8540. 10.1021/bi027363r. 12859200

[ref65] Jeppesen M. D.; Westh P.; Otzen D. E. The role of protonation in protein fibrillation. FEBS Lett. 2010, 584, 780–784. 10.1016/j.febslet.2010.01.002. 20067793

[ref66] Diaferia C.; Morelli G.; Accardo A. Fmoc-diphenylalanine as a suitable building block for the preparation of hybrid materials and their potential applications. J. Mater. Chem. B 2019, 7, 5142–5155. 10.1039/C9TB01043B. 31380554

[ref67] Tang C.; Smith A. M.; Collins R. F.; Ulijn R. V.; Saiani A. Fmoc-Diphenylalanine Self-Assembly Mechanism Induces Apparent pK a Shifts. Langmuir 2009, 25, 9447–9453. 10.1021/la900653q. 19537819

[ref68] Kanicky J. R.; Poniatowski A. F.; Mehta N. R.; Shah D. O. Cooperativity among Molecules at Interfaces in Relation to Various Technological Processes: Effect of Chain Length on the pKa of Fatty Acid Salt Solutions. Langmuir 2000, 16, 172–177. 10.1021/la990719o.

