# Supplementary material for: Charge Regulation during Amyloid Formation of α-Synuclein
Source: J Am Chem Soc. 2021 May 17;143(20):7777–91. doi: 10.1021/jacs.1c01925 (PMC8161422; doi:10.1021/jacs.1c01925)
Supplement: Supplementary file 1 — ja1c01925_si_001.pdf [file ja1c01925_si_001.pdf]

Supporting information

Charge regulation during amyloid formation of α-synuclein

Tinna Pálmadóttir[1*], Anders Malmendal[2], Thom Leiding[1], Mikael Lund[3,4], Sara Linse[1*].

[1] Department of Biochemistry and Structural Biology, Lund University, Sweden.
[2] Department of Science and Environment, Chemistry, Roskilde University, Denmark.
[3] Department of Theoretical Chemistry, Lund University, Sweden.
[4] LINXS - Lund Institute of advanced Neutron and X-ray Science, Lund University, Sweden

[*] E-mail: sara.linse@biochemistry.lu.se
[*] E-mail: tinna.palmadottir@biochemistry.lu.se

Experimental Section

Cloning of α-synuclein and α-synuclein 5Q-mutant

The gene-sequences were cloned in a Pet3a plasmid between NdeI and BamHI with *E. coli*-optimized codons (purchased from GenScript, Piscataway, New Jersey). Note that the ATG in the beginning codes for the starting Met and is part of the NdeI recognition site. Two stop codons were added to appear before the BamHI site. Each mutation adds one or two base changes to the previous gene, marked in yellow. In the following we have changed GAA (E) to CAG (Q) to get high expression.

The DNA sequence for wild-type α-synuclein

5'ATGGACGTTTTCATGAAAGGTCTGTCTAAAGCTAAAGAAGGTGTTGTTGCTGCTG
CTGAAAAAACCAAACAGGGTGTTGCTGAAGCTGCTGGTAAAACCAAAGAAGGTGT
TCTGTACGTTGGTTCTAAAACCAAAGAAGGTGTTGTTCACGGTGTTGCTACCGTTG
CTGAAAAAACCAAAGAACAGGTTACCAACGTTGGTGGTGCTGTTGTTACCGGTGTT
ACCGCTGTTGCTCAGAAAACCGTTGAAGGTGCTGGTTCTATCGCTGCTGCTACCGG
TTTCGTTAAAAAAGACCAGCTGGGTAAAAACGAAGAAGGTGCTCCGCAGGAAGGT
ATCCTGGAAGACATGCCGGTTGACCCGGACAACGAAGCTTACGAAATGCCGTCTG
AAGAAGGTTACCAGGACTACGAACCGGAAGCTTAATAG

to make the following protein sequence:

MDVFMKGLSKAKEGVVAAAEKTKQGVAEAAGKTKEGVLYVGSKTKEGVVHGVATV
AEKTKEQVTNVGGAVVTGVTAVAQKTVEGAGSIAAATGFVKKDQLGKNEEGAPQEG
ILEDMPVDPDNEAYEMPSEEGYQDYEPEA

The DNA sequence for 5Q α-synuclein mutant

5'ATGGACGTTTTCATGAAAGGTCTGTCTAAAGCTAAAGAAGGTGTTGTTGCTGCTG
CTGAAAAAACCAAACAGGGTGTTGCTGAAGCTGCTGGTAAAACCAAAGAAGGTGT
TCTGTACGTTGGTTCTAAAACCAAAGAAGGTGTTGTTCACGGTGTTGCTACCGTTG
CTGAAAAAACCAAAGAACAGGTTACCAACGTTGGTGGTGCTGTTGTTACCGGTGTT
ACCGCTGTTGCTCAGAAAACCGTTGAAGGTGCTGGTTCTATCGCTGCTGCTACCGG
TTTCGTTAAAAAAGACCAGCTGGGTAAAAACGAAGAAGGTGCTCCGCAGGAAGGT
ATCCTGCAGGACATGCCGGTTGACCCGGACAACCAGGCTTACCAGATGCCGTCTGA
AGAAGGTTACCAGGACTACCAGCCGCAGGCTTAATAG

to make the following protein sequence

MDVFMKGLSKAKEGVVAAAEKTKQGVAEAAGKTKEGVLYVGSKTKEGVVHGVATV
AEKTKEQVTNVGGAVVTGVTAVAQKTVEGAGSIAAATGFVKKDQLGKNEEGAPQEG
ILQDMPVDPDNQAYQMPSEEGYQDYQPQA

(mutations: E114Q+E123Q+E126Q+E137Q+E139Q)

Results

Effect of protein concentration on pH and ΔpK_a^{ave}

The effect of increased protein concentration was measured and calculated for both wild-type α-synuclein and α-synuclein 5Q-mutant. The pH change, pK_a^{ave} and ΔpK_a^{ave} for wild-type and 5Q mutant at 20 μM and 70 μM are shown in Table S1.

Table S1: Effect of protein concentration on pH and ΔpK_a^{ave}. Values were calculated for both 20 μM and 70 μM α-synuclein and α-synuclein 5Q-mutant.

	Start pH	*End pH*	ΔpH	*Start pKa*	pK_a^{ave}	ΔpK_a^{ave}
Wt – 20 uM	5.6	6.5	0.9	4.3	5.4	1.1
Wt – 70 uM	5.5	6.6	1.1	4.3	5.5	1.2
5Q – 20 uM	6.4	6.6	0.2	4.2	4.7	0.5
5Q – 70 uM	6.4	7.1	0.7	4.2	5.1	0.9

As can been seen from Table S1, the effect of pH is amplified at higher protein concentration; however, the effect on the pK_a^{ave} values of the wild-type remains similar. However, the effect on the pH and pK_a^{ave} values for the 5Q mutant is slightly higher at higher concentration. We speculate that this could possibly be explained by the packing of the fibrils. The mutant contains five less charges than the wild-type, which presumably causes less repulsion between single fibrils, allowing denser packing of fibrils, leading to increased effect on the pKa values of the fibrils.

Simultaneous measurements of the pH, ThT fluorescence and light-scattering

Simultaneous measurements of the pH, ThT fluorescence and light-scattering was were performed the same sample of wild-type α-synuclein in water (Figure S1). The data shows that the onset of the pH-increase coincides with the onset of the ThT fluorescence and scattering.

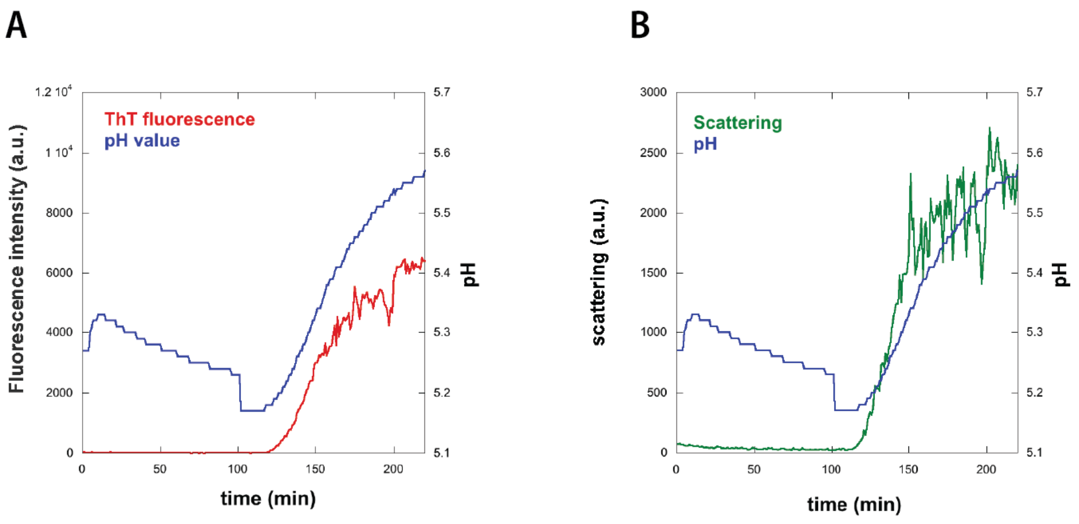

Figure S1: Simultaneous measurements of the pH, ThT fluorescence and light-scattering. 35 µM wild-type α-synuclein was prepared in water. The ThT fluorescence and the scattering was recorded at the same time as the pH value, using the Probe Drum instrument. **A)** The pH and the ThT fluorescence. **B)** The pH and the static light scattering.

Analysis of monomeric concentration remaining in solution after fibril formation in water

The monomeric concentration remaining the fibrillar samples was measured as explained in the experimental section (2.7.) Figure S2 shows an SDS-PAGE of the supernatant of both α-synuclein wild-type (wells: 6-9) and 5Q mutant (wells 3-5), as well as known concentrations of monomeric samples (wells 2; 9-14). This gels was analyzed using the program ImageJ in order to calculate the concentration of monomer remaining in solution, by comparing the intensity of the bands appearing for 5Q mutant and wild-type to the 6 μM monomeric wild-type α-synuclein. The monomeric concentration in 20 μM fibrillar wild-type sample was calculated to be 1.7 μM and in the fibrillar 5Q-mutant sample to be 0.2 μM.

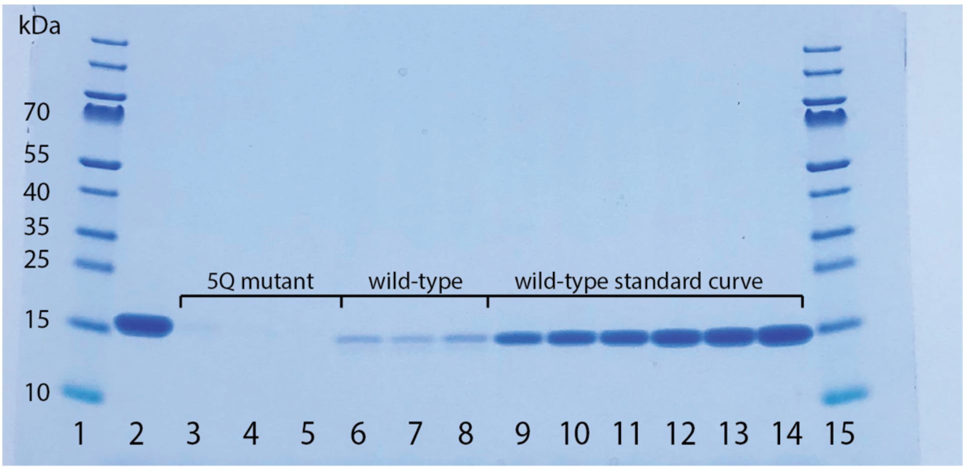

Figure S2: SDS-PAGE containing supernatants of α-synuclein wild-type and 5Q mutant. Two-step separation of fibrils and monomers remaining in solution was performed as explained in section 2.7. Well 1: PageRuler™; well 2: 20 μM 5Q mutant; wells 3-5: supernatant of 5Q mutant after second centrifugation, three replicates of same kind of sample, forming fibrils independently in separate tubes; wells 6-8: supernatant of wild-type after second centrifugation, three replicates of same kind of sample, forming fibrils independently in separate tubes; well 9: 6 μM wild-type; well 10: 8 μM wild-type; well 11: 10 μM wild-type; well 12: 12 μM wild-type; well 13: 16 μM wild-type; well 14: 20 μM wild-type, well 15: PageRuler™.

CD spectroscopy was used to verify the presence of fibrils in the wild-type and 5Q mutant (Figure S3).

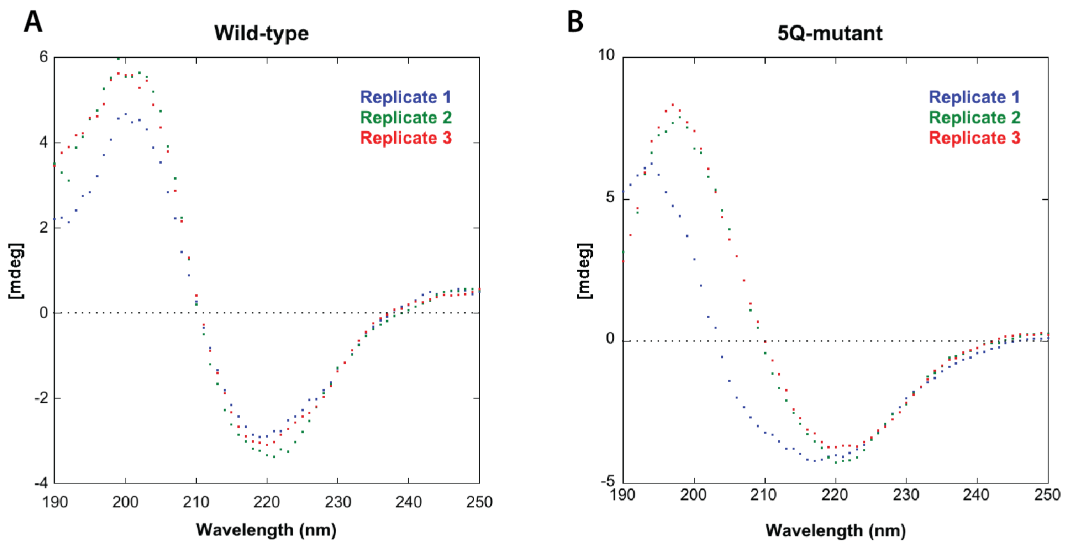

Figure S3: CD spectroscopy of α-synuclein wild-type and 5Q mutant formed in water. CD-spectra were recorded after fibril formation to verify the presence of fibrils in all samples, before the samples were centrifuged for further analysis. The graphs include 3 replicates of each type of sample (A and B), replicates were formed independently in separate tubes. **A)** wild-type **B)** 5Q-mutant

CD-Spectroscopy was used to verify that separation between fibrils and monomers was sufficient. Figure S4 shows a CD of supernatant after first centrifugation; supernatant after second centrifugation (used for the SDS-PAGE); and resuspended pellet after first centrifugation (resuspended in the remaining 150 μL).

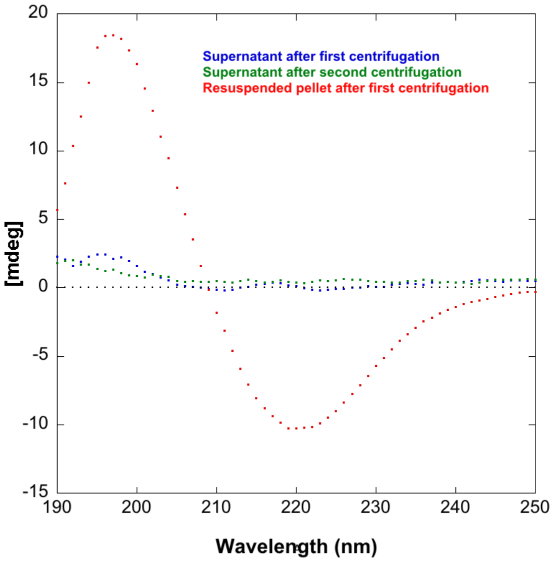

Figure S4: CD-spectroscopy of supernatants and resuspended pellet. Blue: supernatant after first centrifugation; Green: supernatant after second centrifugation; Red: resuspended pellet.

Figure S4 shows that separation of monomers from fibrils in the fibrillar sample was successful. The flat signal of the monomeric supernatant can be explained by low protein concentration, as can be seen from the gel in Figure S2.

Metropolis Monte Carlo simulations of α-synuclein monomer and fibril at pH 5.5

Ionisation states of α-synuclein monomer and fibril (represented by ten planes, PDB: 2N0A) were studied using constant pH Metropolis Monte Carlo simulations. Figure S5 shows the differences in residue partial charges of α-synuclein monomer and fibril, calculated for both the wild-type α-synuclein and the α-synuclein 5Q-mutant at constant pH 5.5 (see Figure S5).

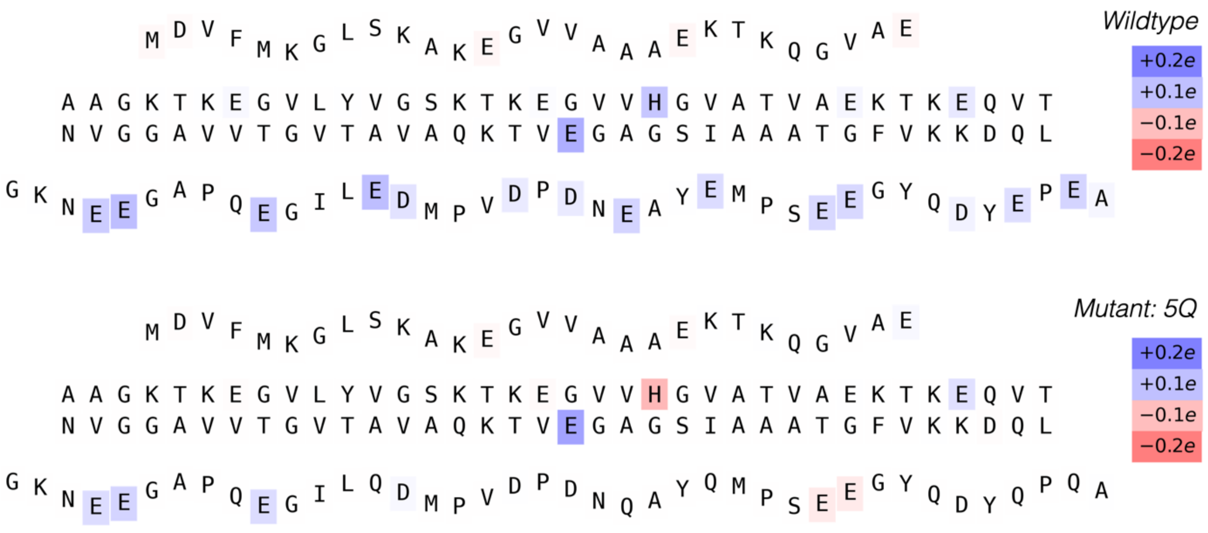

Figure S5. Metropolis Monte Carlo simulations of α-synuclein monomer and fibril at pH 5.5 (10 planes of a fibril as found in PDB: 2N0A). Differences in residue partial charges between α-synuclein monomer and fibril at constant pH 5.5, calculated for both the wild-type α-synuclein and the α-synuclein 5Q-mutant. Shifts (charge in fibrillar minus charge in monomeric state) are indicated by the colourbars and letters correspond to the amino acid sequence with a rigid middle part surrounded by flexible (shown in “wavy” text) N-terminal and C-terminal parts.

Fluctuations of average upshift in pKa values during fibril formation due to increase of monomeric pKa values.

The fluctuations in average upshift of pK_a values during fibril formation due to higher monomeric pK_a values were calculated (Figure S6). The monomeric pK_a values were increased step-wise on average from 0.1 to 1. This was done both by only taking into account the pK_a increase of the acidic residues in the C-terminal tail and also by averaging the upshift of pK_a over all acidic residues within the protein sequence. This showed that the average pK_a upshift of fibrils is not greatly affected by higher monomeric pK_a values.

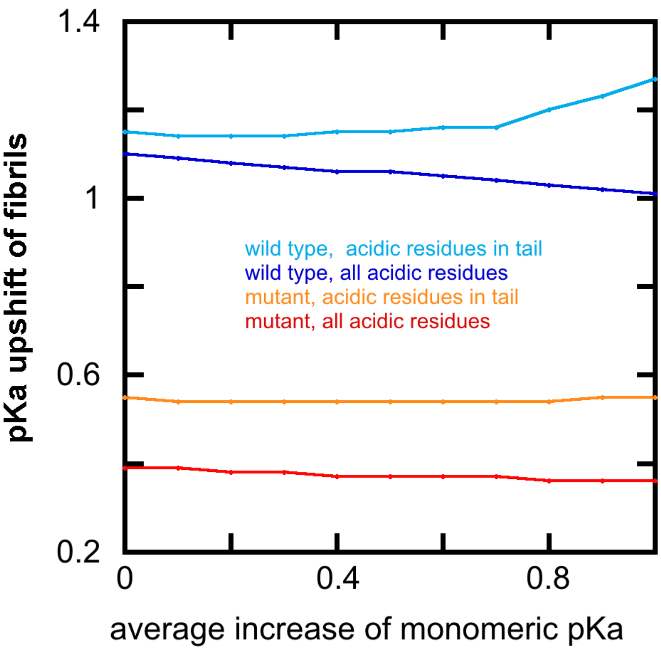

Figure S6: Fluctuations of average upshift in pK_a values during fibril formation due to increase of monomeric pK_a values.

Protein Net-charge from Metropolis Monte Carlo Simulations

Figure S7 shows the simulated total charge of monomeric and fibrillar (decameric) α-synuclein between pH 2 and pH 8. The pH titration curves for monomeric α-synuclein give a nearly symmetrical curve. However, for the fibril the titration curves are more stretched out and it shows that the fibril does not reach full protonation at pH 2. This can be explained by the strong positive potential from the cationic fibril, opposing the protonation of the acidic groups, resulting in lower net charge.

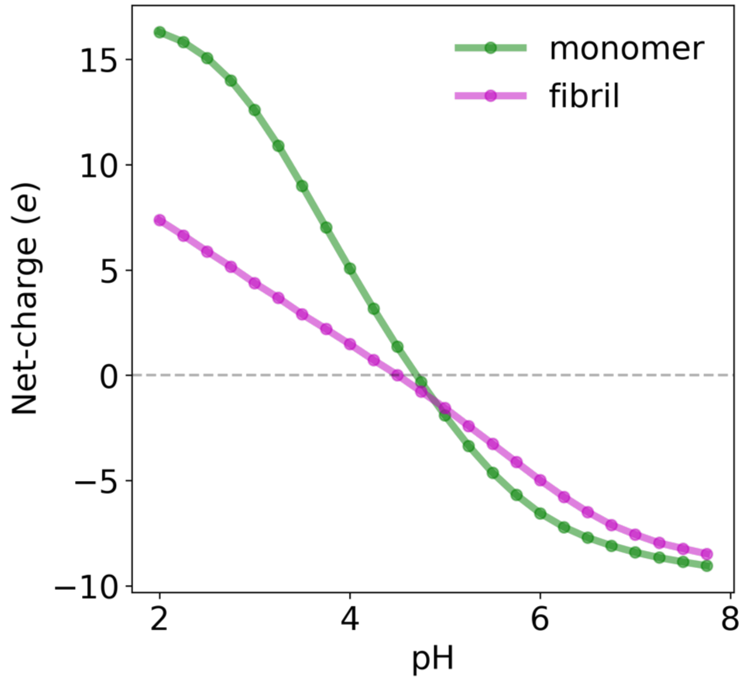

Figure S7: Simulated total charge of α-synuclein in monomers (green) and when embedded in a fibril (magenta).
